# Export of macroinvertebrate prey from tidal freshwater wetlands provides a significant energy subsidy for outmigrating juvenile salmon

**DOI:** 10.1371/journal.pone.0282655

**Published:** 2023-03-17

**Authors:** G. Curtis Roegner, Gary E. Johnson

**Affiliations:** 1 Northwest Fisheries Science Center, National Marine Fisheries Service, National Oceanic and Atmospheric Administration, Point Adams Research Station, Hammond, Oregon, United States of America; 2 Coastal Sciences Division, Pacific Northwest National Laboratory, Portland, Oregon, United States of America; King’s College London, UNITED KINGDOM

## Abstract

Tidal freshwater wetlands linking terrestrial, riverine, and saline habitats are critical areas for material processing and exchange. Once historically widespread, herbaceous marsh and forested tidal freshwater wetlands especially are now highly degraded worldwide. Additionally, quantitative assessments of hydrology and material exchange from these systems are lacking compared to lotic and estuarine (saltmarsh) habitats. Here we investigate macroinvertebrate and energy export from tidal marsh and forested wetlands and consider potential benefits from this ecological process to endangered Pacific salmon in a large tidal freshwater system, the Columbia River (USA). Macroinvertebrate (salmon prey) concentration, water velocity, and discharge were measured at several wetland habitat types (forested swamp, emergent marsh, and restored marsh). We used these data to compute prey flux and transport metrics. Then, applying literature values to calculate prey energy equivalents and juvenile salmon metabolic requirements, we estimated the potential energy subsidy available to juvenile salmon. Numerically, larval stages of aquatic insects were the predominant type of prey exported from the wetlands, with Diptera chironomid fly abundance exceeding other groups. Energetically, however, non-chironomid dipterans and hemipteran prey comprised most of energy transport due to their higher energetic content (energy density × mean weight). We determined the prey energy transported from the sampled tidal channels was sufficient to meet energetic needs of tens to thousands of juvenile salmon per day, depending on prey production and hydrography. The prey taxonomic composition differed among organisms exiting forested swamp, emergent marsh, and restored marsh habitats with corresponding differences in energy transport, but all habitat types supported similar numbers of juvenile salmon. We conclude that macroinvertebrate prey exported from varied tidal freshwater wetlands likely provide significant benefits to juvenile salmon over a larger ecological footprint than the wetland area would suggest.

## Introduction

Tidal wetlands are key centers of matter and energy transformation in aquatic systems [1, 2]. Their degradation worldwide has altered numerous ecosystem functions including nutrient cycling [3], carbon sequestration [4, 5], and reduction of critical habitat for a diversity of organisms [6] such as imperiled Pacific salmon [7]. A fundamental attribute of well-functioning tidal wetland systems is the export of organic production to adjacent environments via tidal channel pathways [8–10]. Exported material can include dissolved, particulate, or living matter that can be converted into energy equivalents [9]. This study concerns energy exported from tidal wetlands via macroinvertebrate prey for juvenile salmon.

Wetlands can be both source and sink for organic matter. To evaluate wetland productivity, studies often estimate net values (source/sink) of materials by differencing flood tide transport from ebb transport [11]. With respect to macroinvertebrate prey, these tidal transports are determined by integrating instantaneous prey flux (prey/m^2^/s) with channel cross-sectional area to yield prey transport (prey/s). Instantaneous transport is integrated over tidal time periods (flood or ebb) to yield total flood or ebb transport (prey/tide). Our focus is on export rather net productivity, and we use concurrent measurements of prey concentration, water velocity, and discharge during ebb tides to characterize prey taxa (and energy) moving from wetlands to deeper channel habitats.

Theoretical and empirical studies of material fluxes and transports have been conducted for decades in estuarine saltmarshes [2, 12–14]. However, there has been much less attention to material transport from tidal freshwater (TFW) habitats, which are elemental to the fluvial-estuarine continuum of tidal rivers [1, 15, 16]. Past research has revealed many important aspects of material movements and transformations in tidal wetland systems, including focuses on physical forcings [6], dissolved and particulate organic matter [9, 17, 18], sediment budgets [13, 19, 20], phyto- and zooplankton movements [21–24], larval recruitment of invertebrates [25–27], and fish habitat use [28, 29]. There has also been considerable interest in material transport in lotic systems, where researchers have measured the contribution of terrestrial and aquatic vertical fluxes (inputs and exports) as subsidies to diets of salmonids and other fishes [30, 31]. In some lotic systems, researchers quantified the magnitude of invertebrate “drift”, the downstream flux or transport of potential prey [32–34]. In contrast, few studies in tidal systems concentrated on the transport of non-planktonic macroinvertebrates, such as aquatic and terrestrial insects, arachnids, and epibenthic amphipods. Many of these taxa have life-history stages dependent on wetlands or riparian zones, yet are found in stomach contents of consumers outside wetland habitats in the mainstem river [35, 36]. Tidal transport of aquatic and terrestrial macroinvertebrate prey constitutes an indirect benefit (subsidy) from wetlands to the wider ecosystem [31], but the magnitude of this subsidy in TFW systems is largely unknown.

Here, we apply concepts of flux and transport originally developed for saltmarsh systems, along with considerations of invertebrate prey subsidies derived from lotic systems, to evaluate ebb flux and transport of macroinvertebrate prey (and energy) from TFW wetlands in the lower Columbia River and estuary (LCRE). This highly regulated river system once supported extensive TFW habitats extending from near the estuary mouth to over 235 km upstream [37, 38]. To remedy this loss, habitat restoration activities in the LCRE over the last several decades have emphasized increasing hydrological connectivity to enhance opportunity for juvenile salmon to access wetland interiors, as well as enhance export of prey material to the mainstem [39–41].

One driver of restoration activities in the LCRE is the listing under U.S. Endangered Species Act of four of the five Pacific salmonid species in the Columbia River basin: *Oncorhynchus keta* (Chum Salmon), *O*. *mykiss* (steelhead), *O*. *nerka* (Sockeye Salmon), and *O*. *tshawytscha* (Chinook Salmon). Research has demonstrated fundamental differences in habitat preferences among these species and life history patterns within a species [42, 43]. For example, many of the smaller (<100 mm fork length) subyearling Chinook and Chum Salmon extensively reside and feed within wetland tidal channels and shallow water areas. Conversely, larger fish such as yearling Chinook and Coho (*O*. *kisutch*) and steelhead emigrate to the ocean more rapidly and are less affiliated with shallow habitats than smaller juvenile salmonids. However, diet analyses from sampling in the mainstem LCRE show these larger yearling fish commonly ingest insects and other prey that originate primarily in floodplain wetlands [35, 36]. The source of these mainstem prey items is of particular importance to restoration and conservation actions. Accordingly, we hypothesized a primary prey source for larger yearling fish in the mainstem is macroinvertebrates exported from wetland sites during ebb tide flows.

The objectives of our study are twofold. First is to quantify the type, composition, flux, and ebb transport of macroinvertebrate prey from intertidal channels for several wetland habitat types. The second objective is to use simple bioenergetic considerations to evaluate the magnitude of prey energy transport as a wetland energy subsidy to juvenile salmon elsewhere in the system. While our focus is on prey export from TFW wetlands, the methods and results apply to tidal wetlands in general worldwide.

## Materials and methods

### Study setting

**Fig 1:**
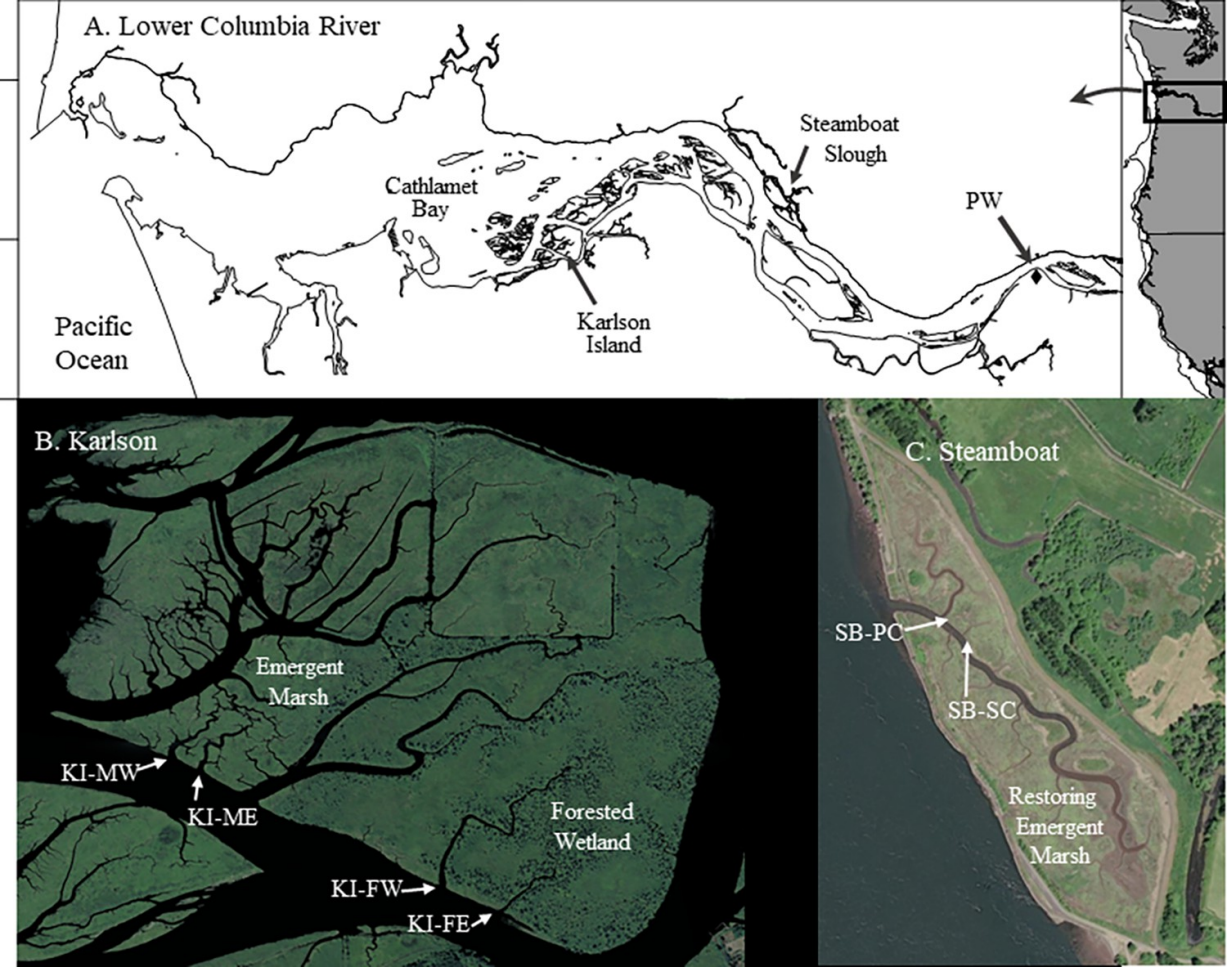
A. Location of study areas and sampling sites in the LCRE. B. Karlson Island. C. Steamboat Slough.

We sampled three representative tidal freshwater habitat types: forested swamp, emergent marsh, and restored emergent marsh (Fig 1). The sites varied in cross-sectional area, depth, and bankfull area (Table 1). The forested sites were Sitka spruce (*Picea sitchensis*) swamp (Forested) located on Karlson Island and emergent herbaceous marshes (Marsh) at Karlson Island and Steamboat Slough (Restored). Karlson Island (river kilometer 40) is a complex site containing reference emergent and forested wetlands (Fig 1), as well as a region where a levee was breached by natural processes in the late 1960s. Emergent marshes in this area resemble undisturbed portions of the wetland complex and nearby sites. Steamboat Slough (river kilometer 57) was the site of a tidal reconnection project completed in 2014. At all sites, sampling occurred during ebb tides at the mouths of tidal channels (“sloughs”) that drain into larger channels that remain subtidal. We sampled at three tidal channels during May–July 2016 and six during April–June 2017. The 2016 samples comprised a pilot study (nine sampling dates) to develop methods and determine the taxonomic composition of exported prey. We then conducted a focused examination of macroinvertebrate flux and transport during 2017 (19 sampling dates). Here we emphasize results from the 2017 field work.

**Table 1:** Morphological characteristics at the wetland channel sampling sites during 2017. Area, depth, width, and the width:depth ratio (W:D) were measured at the site of the ADCP deployment. Bankfull surface area and perimeter values were obtained from Google Earth (ver. 7.3.3).

Habitat	Station	Area (m^2^)	Depth (m)	Width (m)	W:D	Bankfull area (m^2^)	Perimeter (m)
**Forest**	KI-FE	40	3.4	21	6.2	5229.3	948.0
	KI-FW	56	3.0	28	9.3	14602.0	2352.4
**Marsh**	KI-ME	28	1.8	25	14.3	6912.1	1233.8
	KI-MW	32	1.5	26	17.3	6939.9	1269.0
**Restored**	SB-PC	16	1.3	13	10.0	4513.5	1439.4
	SB-SC	9	1.2	6	5.0	284.0	159.4

### Water velocity and discharge

Quantifying the horizontal flow of matter and energy in tidal channels has instantaneous and net components [11]. Since flow in tidal systems is unsteady, accurate time series data are required to calculate fluxes and transports. We used a bottom mounted acoustic Doppler current profiler (ADCP, SonTek IQ) to measure ebb velocity (*U*, m/s) and discharge (*Q*, m^3^/s) from the mouths of wetland tidal channels. The instrument uses acoustic beams at 3.0 MHz to profile velocities fore, aft, and lateral to the flow direction. A fifth vertical beam and a pressure sensor both measure depth. Discharge was calculated from the resultant index velocity, water depth, and surveyed channel cross-sectional area [44, 45]. During deployment, the ADCP was attached to a weighted plate and leveled on the tidal channel substrate. Velocity and water level measurements were made at 60-s intervals, and mean velocity and discharge were computed, averaged, and recorded every 5 or 10 min (depending on sampling date). For convention, we assigned a positive value to ebb velocity and discharge. (Note this convention differs among studies in the literature.)

### Prey composition

At the same location as for ADCP sampling, we sampled plankton and drift organisms with a 1-m long, 300 μm mesh neuston net equipped with a calibrated flow meter (General Oceanics). For a given sample, the net was hauled across the channel on a pulley system 1–3 times over a 3–5 min period, yielding a mean filtered volume of 9.9 ± 3.9 m^3^ (±standard deviation, SD). Samples were collected at approximately 0.5-h intervals from bankfull stage until the water level decreased to approximately 0.3 m, when it became too shallow to deploy the net. Discharges were negligible (< 0.05 m^3^/s) during these low water levels. We discarded samples contaminated by bottom sediments. Sampling was thus restricted to a portion of the tidal range with no sampling conducted during either overbank or very shallow water levels. The macroinvertebrates we collected were preserved in 70% ethanol. No permits were required to sample macroinvertebrates. In the laboratory, organisms were identified to the lowest possible taxon, then sorted into prey categories as described below.

We used data from both 2016 and 2017 to identify major taxa exiting tidal creeks across study sites. For further analysis, we then categorized prey taxa based on the overall abundance and frequency of occurrence, the prevalence of taxa in salmon diets from previous studies in the LCRE, and whether the taxon likely originated within the sampled wetland complex [35, 42, 46]. We ignored planktonic taxa (copepods and cladocerans) that are flushed between intertidal wetlands and subtidal channel habitats, as well as several benthic taxa (ostracods, oligochaetes, and nematodes) that are not necessarily tied to wetlands. Relative abundances of these groups are included for reference as shown below. Epibenthic-pelagic mysids were included due to their large size (energy content) and prevalence in salmon diets [42]. We categorized the remaining prey data into the following groups: arachnids composed of Acari (mites) and Arenaea (spiders); malacostracans including Amphipoda and Mysida; and the Insecta. We further divided the Insecta order Diptera (flies) into the families Ceratopogonidae (non-biting midges) and Chironomidae (biting midges), and Other Diptera. The order Hemiptera (true bugs) was divided into the families Aphididae (aphids) and Corixidae (water boatmen), and Other Hemiptera. A final category was Other Insecta, composed of orders Coleoptera, Collembola, Ephemeroptera, Hymenoptera, Lepidoptera, Megaloptera, Odonata, Psocoptera, Thysanoptera, and Trichoptera. All these macroinvertebrate taxa are known prey items for juvenile salmon [42, 46].

### Prey concentration, flux, and transport

For each plankton net sample, we determined the total concentration of prey (*C*_*T*_, ind/m^3^) and the proportional composition of each taxa by dividing the count of individual prey by the volume of water sampled by the net. Instantaneous prey flux (*F*_*I*_, ind/m^2^/s) was calculated by pairing mean channel velocity (*U*, m/s) measured concurrently during net sampling with the corresponding total prey concentration (*C*_*T*_), as follows:

$$F_{I}=C_{T}\times U.$$

Similarly, instantaneous prey transport (*T*_*I*_; ind/s) was computed by pairing mean discharge (*Q*, m^3^/s) with total prey concentration for each net sample, as follows:

$$T_{I}=C_{T}\times Q.$$

For each sampling date, we integrated instantaneous prey transport for a given taxa (*T*_*ITAXA*_) over time to yield total ebb transport (*T*_*TAXA*_, ind/ebb tide). Since the time steps for the water discharge time series were measured at a higher frequency (5- or 10-min intervals) than for the prey concentration time series (~30 min intervals), we interpolated over the discharge measurement time steps for the integration, as follows:

$$T_{TAXA}=\int T_{ITAXA}dt$$

Finally, we summed the transport for all taxa (*p* is number of taxa) to yield total transport (*T*, ind/ebb tide), as follows:

$$T=\sum_{j=1}^{p}T_{TAXA_{j}}$$

An example of how time series data for prey and water were used for transport calculations is provided in S1 Fig in S1 Appendix, where we calculated instantaneous flux and transport rates using total prey and chironomid concentration data.

For data presentation, we report total ebb transport and the percent contribution of each taxa category to total transport and, for each sampling date, the percent of the tide sampled and the mean (± standard error) values of *C*_*T*_, *U*, *Q*, *F*_*I*_, and *T*_*I*_. We also calculated the total ebb discharge volume (*D*, m^3^). To examine the composition of prey within the total transport, we plotted the percent of each taxa of interest by observation period and habitat type.

### Wetland energy subsidy

We investigated the potential benefit of the invertebrate prey as a subsidy to juvenile salmon by comparing prey energy transport to the daily metabolic needs of a yearling Chinook Salmon. Prey taxa energy content (*EC*, J/ind) was determined as the product of prey energy density (*ED*, J/mg) and prey wet weight (*W*, mg), as follows:

EC=ED×W.

Energy density values were obtained from bomb calorimetry measurements [46, 47]. Mean prey wet weights were derived from salmon stomach contents [48] (Table 2).

**Table 2:** Prey taxa mean weights (*W*; mg wet weight) and statistics, and values of energy density (*ED*, J/mg) and energy content (*EC*; J/ind).

Class	Taxa Group	n	W^a^	SD	-95% CI	+95% CI	*ED* ^b^	*EC*	*EC*_*REL*_
**Arachnida**	Acari	4	0.06	0.04	-0.01	0.13	5.32	0.3	0.2
	Araneae	46	1.82	3.1	0.90	2.74	5.32	9.7	6.1
**Malacostraca**	Amphipoda	144	2.61	3.2	2.08	3.13	3.00	7.8	4.9
	Mysidae	4	17.58	13.2	-3.43	38.58	3.55	62.4	39.0
**Insecta**	Ceratopogonidae	44	3.44	10.66	0.20	6.69	3.83	13.2	8.3
	Chironomidae	375	0.43	0.52	0.38	0.48	3.83	1.6	1.0
	Diptera	173	1.26	4.75	0.55	1.98	8.92	11.3	7.1
	Aphididae	49	0.41	0.23	0.34	0.47	10.93	4.4	2.8
	Corixidae	25	2.85	2.21	1.94	3.77	10.93	31.2	19.5
	Hemiptera	24	1.45	1.43	0.84	2.05	10.93	15.8	9.9
	Other Insecta	243	2.90	8.66	1.81	4.00	7.48	21.7	13.6

n, number of individuals weighed. SD, standard deviation. CI, confidence interval. *EC*_*REL*_ is *EC* scaled to (i.e., divided by) chironomid energy content.

^a^[48]

^b^[46, 47].

Energy transport for each prey taxa (*ET*_*TAXA*_) was computed by pairing transport and energy content, as follows:

$$ET_{TAXA}=T_{TAXA}\times EC_{TAXA}.$$

Summing *ET*_*TAXA*_ over all taxa produced total energy transport per tide (*ET*_*TOT*_, kJ/ebb tide), as follows:

$$ET_{TOT}=\sum_{j=1}^{p}ET_{TAXA_{j}}.$$

In this manner, we determined the proportion of each taxa contributing to the total amount of energy exported and available as a subsidy for juvenile salmon and their food webs. We tabulated *ET*_*TOT*_ for each sampling date and plotted the proportion of each taxa contributing to the total energy exported for each habitat type.

**Fig 2:**
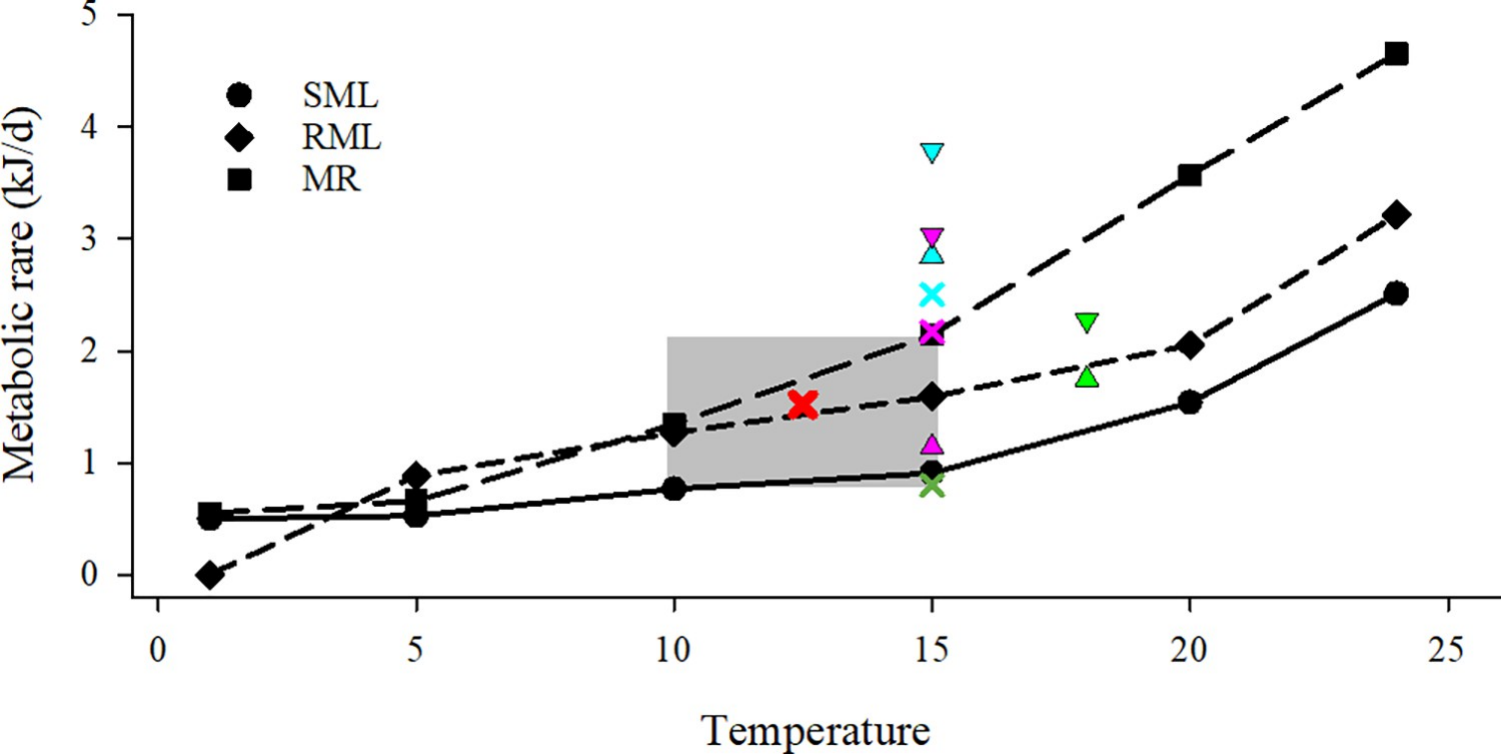
Calculated metabolic rates in relation to temperature for a 155 mm (0.038 kg) juvenile salmon. This equates to an energy requirement of 40 kJ/kg/d (40 = 1.52 / 0.038). Lines: Standard metabolic level (SML), routine metabolic level (RML), and maintenance ration (MR) for Sockeye Salmon (black lines and symbols [50]). Shaded area denotes rates within the 10–15°C range. Symbols: SML for rainbow trout: blue triangle [51]; green triangle [53]; green cross [54]; blue cross [55]. SML for Atlantic Salmon: pink cross [56]. MR for rainbow trout: blue triangle [51]. Up and down triangles represent range of values. Red X, metabolic rate used in estimation.

To estimate the potential benefit of the prey export to juvenile salmon, we compared total ebb transport energy values to daily energy requirements for a standard juvenile salmon (*ER*_*S*_; kJ/kg/d). The metabolic rate value is an estimate of the energy-equivalent prey ration necessary to meet the daily energetic requirement for a fish with minimal activity [49]. Based on literature values, we used an energy requirement of 40 kJ/kg/d for juvenile salmon (Fig 2). This rate falls between the standard metabolic level and the maintenance ration calculated for sockeye salmon across temperatures of 10–15°C [50, 51]. We did not find comparable values for Chinook Salmon in the literature. For comparison among sites, the daily energetic requirement was standardized to the mean size and weight of yearling Chinook Salmon [52]: 155 mm, and 0.038 kg. The daily energetic requirement for this size yearling fish is 1.52 kJ/d. Note a standard-size subyearling [52] (80 mm and 0.005 kg) would require 0.22 kJ/d. Thus, the standard ratio of yearling to subyearling daily energetic requirement is 1:6.9 (1.52/0.22 ≈ 6.9).

By definition, wetland energy subsidy (*WES*, juvenile salmon/ebb tide) is the number of standard juvenile salmon that could be supported by the total prey energy transport for a given tide sampled, calculated as follows:

$$WES=ET_{TOT}/ER_{S}$$

where, *ER*_*S*_ = standard energy requirement for a given juvenile salmon (kJ/fish). Essentially, *WES* is the number of standard juvenile Chinook Salmon that theoretically could be supported by the transported prey energy (Table 2).

As an example using chironomid midges (the numerically dominant prey type), 1 g of exported prey = 3.83 kJ of energy, a level that would support 3.83 / 1.52 = 2.52 yearling salmon (or 17.4 subyearlings). Using these fish and prey parameters and field-collected estimates of prey transport, we calculated the total *WES* from reference and restoration tidal channels and tabulated the results. Note, *ET*_*TOT*_ is the total energy transported in a single ebb tide and there are two unequal ebb tides each day in the LCRE. Therefore, the true number of fish supported on a daily basis is higher than *WES*, but not necessarily double the value due to unequal tidal amplitudes and probable lack of nocturnal feeding for most salmon.

We pooled prey samples collected during May and June (when many yearling Chinook Salmon were migrating [43] to compare the contribution of each taxa to the transport of prey and energy from the three habitats (forested, marsh, and restored marsh)). To highlight the effect of differing prey energy contents on the total energy subsidy, we calculated the proportional anomalies for each taxa as energy transport proportion minus prey transport proportion. High anomalies (outside ± 5%) indicate the degree energy subsidy differed from that expected from numbers transported.

## Results

### Prey composition and energy density

**Fig 3:**
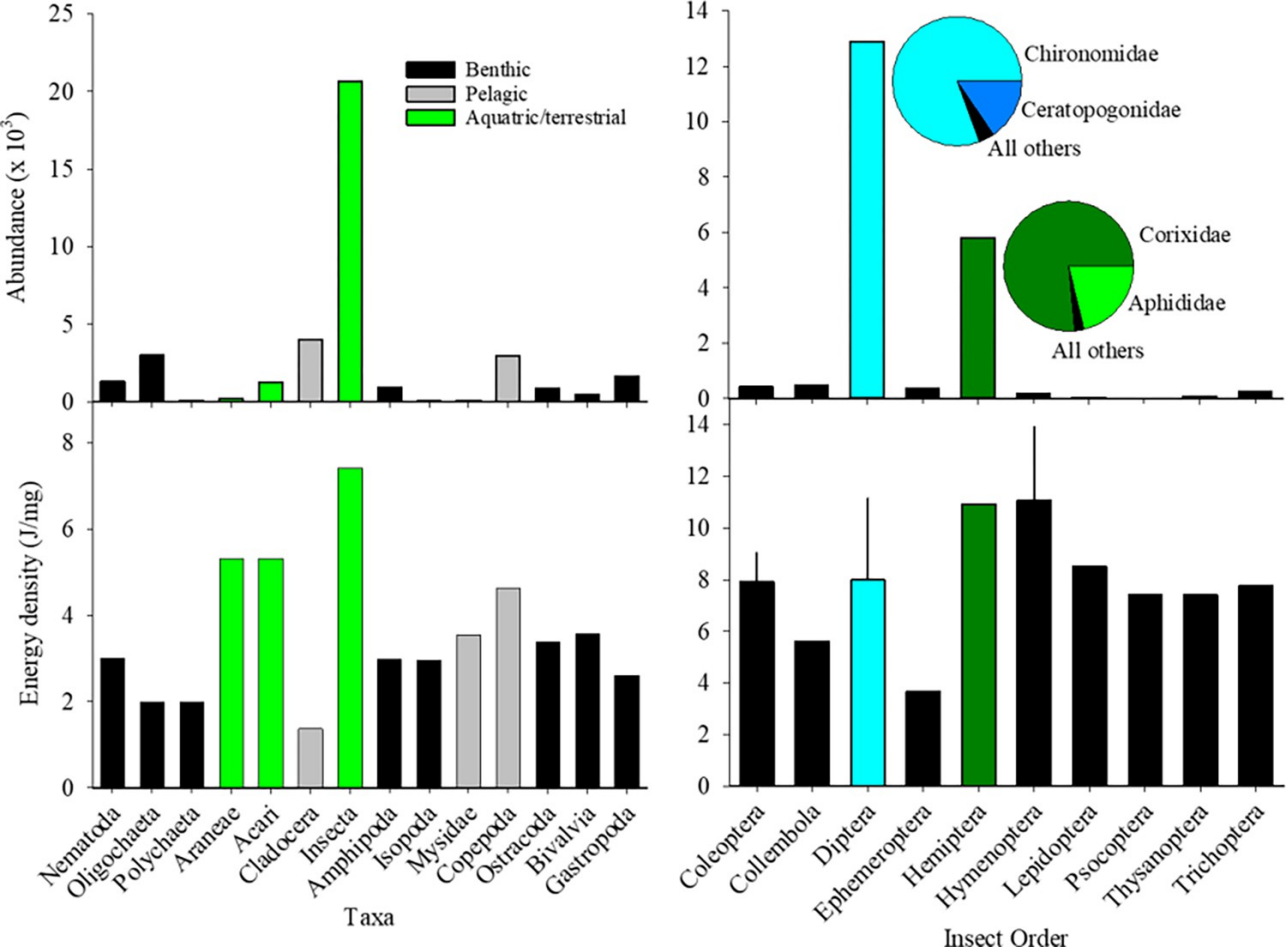
Abundances of taxa (top panels) and corresponding mean energy densities (bottom panels) from plankton samples during 2016 and 2017 combined. Left panels: phyla color-coded for primary habitat. Right panels: Insect orders, highlighting dipterans and hemipterans. Error bars for insect orders are standard deviations where sufficient data were available.

We combined all 28 sampling dates and 288 plankton tows from the 2016 pilot and 2017 main field studies to provide a full accounting of taxa transported out to the tidal creeks. We captured over 63,000 individual macroinvertebrates from 14 major phylogenic groups (Fig **3**). Insects comprised 46% of all individuals with a frequency of occurrence of 99.9% in the tows. Of 12 insect families identified, dipterans and hemipterans comprised 58.1% and 33.3% all of insects with frequencies of occurrence of 98.6% and 61.0%, respectively. The remaining insect families were < 2.0% of total insects. Malacostracans (amphipods, isopods, mysids, copepods, ostracods) comprised 4.2% and arachnids (Araneae and Aacari) 3.1% of the total macroinvertebrates collected. Of the macroinvertebrates captured, more were from aquatic/terrestrial habitats compared to benthic or pelagic habitats. Mean concentration, diversity, and number of taxa differed between habitat types (see S2 Appendix).

**Fig 4:**
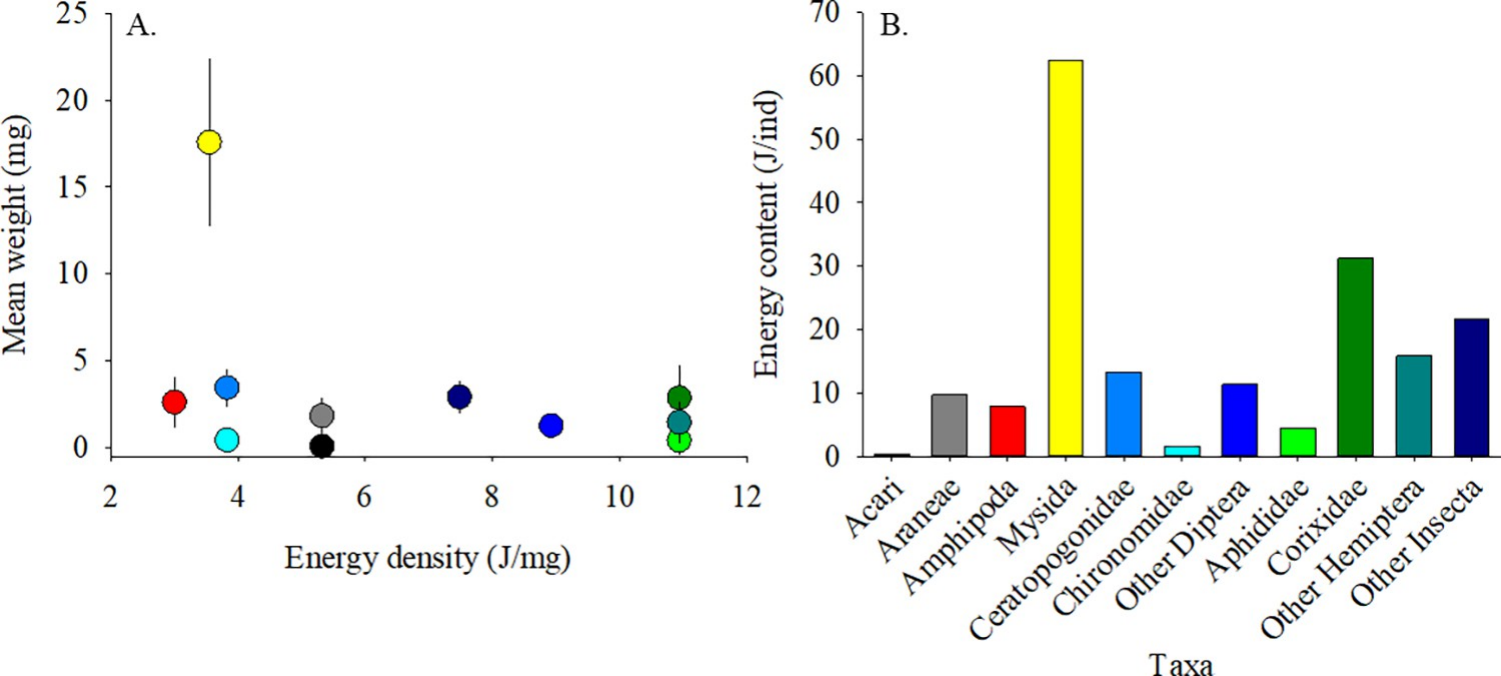
Variation in energy attributes of prey taxa. A. Weight and energy density. Error bars are standard errors. B. Energy content by taxa. Color codes in Fig 4A correspond to the taxa designations in Fig 4B.

Energy densities exhibited no relationship to mean prey weights (Fig 4). However, these variations determined the estimated energy contents (*EC*) of taxa (Table 2; Fig 4). Most insect taxa were of moderate weight (1.3 to 3.4 mg/ind) and high *ED* (7.4 to 10.9 J/mg) resulting in *EC* values > 10 J/ind. The important exceptions were the chironomids (low *EC* due to low *ED* and low *W*) and aphids (low *EC* due to low *W* despite high *ED*). The malacostracans had low *ED*, but with moderate (amphipods) and large (mysids) weights, *EC* values were moderate and very high, respectively. Arachnids were of moderate *ED* but were either very low *W* (Acari) or moderately sized (Araneae), yielding low and moderate *EC*, respectively. Neglecting issues such as digestibility and behavioral selectivity, *EC* values constitute a measure of the quality of taxa as prey to salmonids, with larger prey generally being of higher quality.

### Prey concentration, flux, and transport

All components of flux and transport in tidal streams are time-varying. During 2017, total prey concentration ranged from 0.0 to 79.09 ind/m^3^ with mean values per sample date ranging from 0.66 to 21.71 ind/m^3^ (Table 3). Instantaneous water velocities during prey sampling ranged from <0.01 to 0.25 m/s (mean by sampling date ranged from 0.05 to 0.13 m/s). Instantaneous discharge ranged from 0.01 to 4.9 m^3^/s and tended to peak at the bankfull water level and decline as water levels decreased. Mean discharge by sampling date (0.37 to 3.23 m^3^/s) was positively associated with bankfull area, with the larger wetland systems (e.g., KI-FW, Fig 1 and Table 1) having higher overall discharges than the smaller ones (e.g., SB-SC). Mean instantaneous prey flux ranged from 0.03 to 2.29 ind/m^2^/s. Mean instantaneous transport ranges were 0.57 to 26.95 ind/s (by sample, 0.03 to 60.0 ind/s). Generally, these instantaneous metrics were not statistically associated with stage of tide nor bankfull area (see regression analyses in Supplemental Information). The tidal and areal patterns of total prey concentration, water velocity, and discharge largely determined those for flux and transport.

**Table 3:** Summary variables for 2017 sample dates.

Habitat	Station	DOY	PT (%)	n	*C*_*T*_ (ind/m^3^)	*U* (m/s)	*Q* (m^3^/s)	*F* (ind/m^2^/s)	*T*_*I*_ (ind/s)	*D* × 10^4^ (m^3^)	*T* × 10^5^ (ind)	*ET*_*TOT*_ × 10^3^ (kJ)	*WES* × 10^3^ (fish/ebb tide)
**Forest**	KI-FE-01	94	0.64	8	1.41±0.26	0.08±0.01	1.66±0.35	0.10±0.01	1.89±0.37	2.50	0.30	0.25	0.17
	KI-FE-02	124	0.53	7	1.77±0.62	0.08±0.01	1.48±0.20	0.14±0.05	3.05±1.42	2.08	0.39	0.25	0.17
	KI-FE-03	151	0.71	10	5.17±1.41	0.09±0.02	1.79±0.24	0.34±0.06	8.07±2.13	2.28	1.95	2.17	1.43
	KI-FW-01	95	0.69	9	0.66±0.19	0.07±0.01	1.16±0.26	0.03±0.01	0.57±0.14	1.73	0.08	0.07	0.05
	KI-FW-02	123	0.68	10	1.57±0.40	0.11±0.01	3.23±0.36	0.17±0.03	5.41±1.71	5.21	0.72	0.50	0.33
	KI-FW-03	152	0.69	11	1.96±0.26	0.10±0.01	3.06±0.43	0.20±0.03	6.12±1.18	1.69	0.92	0.89	0.59
**Marsh**	KI-ME-01	96	0.71	9	2.54±2.20	0.10±0.01	1.18±0.28	0.15±0.11	0.99±0.49	--	0.18	0.26	0.17
	KI-ME-02	129	0.71	8	4.08±2.42	0.10±0.01	1.67±0.27	0.22±0.06	4.27±1.39	2.43	0.61	0.53	0.35
	KI-ME-03	150	0.31	5	5.29±1.72	0.09±0.01	0.61±0.22	0.41±0.11	2.15±0.44	0.14	0.18	0.17	0.11
	KI-MW-01	108	0.51	7	21.71±11.26	0.07±0.01	1.04±0.30	1.50±0.75	7.94±2.67	1.08	0.51	0.28	0.18
	KI-MW-02a	122	0.18	4	8.57±6.26	0.11±0.01	1.68±0.38	0.88±0.62	8.16±4.36	0.72	0.43	0.17	0.12
	KI-MW-02b	125	0.39	5	11.79±10.01	0.06±0.01	1.31±0.11	0.92±0.80	13.18±10.81	0.85	0.13	0.13	0.08
	KI-MW-02c	139	0.55	6	18.52±11.65	0.06±0.01	0.84±0.18	1.15±0.72	9.04±3.03	0.83	0.90	0.46	0.30
	KI-MW-03	153	0.77	10	10.72±3.78	0.07±0.01	1.30±0.17	0.76±0.30	10.20±2.08	1.32	1.52	1.05	0.69
**Restor**	SB-PC-02	142	0.90	10	18.44±2.76	0.13±0.02	1.65±0.35	2.29±0.29	26.95±5.75	2.67	4.36	2.09	1.37
	SB-PC-03	170	0.88	9	9.74±1.71	0.11±0.02	1.12±0.22	1.24±0.36	12.98±4.34	1.66	1.83	0.53	0.35
	SB-SC-01	114	0.76	7	2.03±0.58	0.09±0.02	0.60±0.15	0.11±0.03	0.71±0.19	0.56	0.11	0.05	0.04
	SB-SC-02	137	0.67	7	3.87±0.82	0.05±0.02	0.37±0.14	0.25±0.12	1.77±0.90	0.41	0.23	0.07	0.05
	SB-SC-03	171	0.72	8	3.58±0.98	0.09±0.03	0.57±0.17	0.03±0.10	2.02±0.72	0.58	0.22	0.07	0.04

Mean (± SE) prey concentration (*C*_*T*_), velocity (*U*), discharge rate (*Q*), instantaneous flux (*F*), and instantaneous transport (*T*_*I*_). Also included are total ebb discharge volume (*D*), prey transport (*T*), energy transport (*ET*), and wetland energy subsidy (*WES*) for Reference, Marsh, and Restoration (Restor) habitats. DOY, day oy year, PT, percent of tide sampled; n, number of net samples; —, missing data.

**Fig 5:**
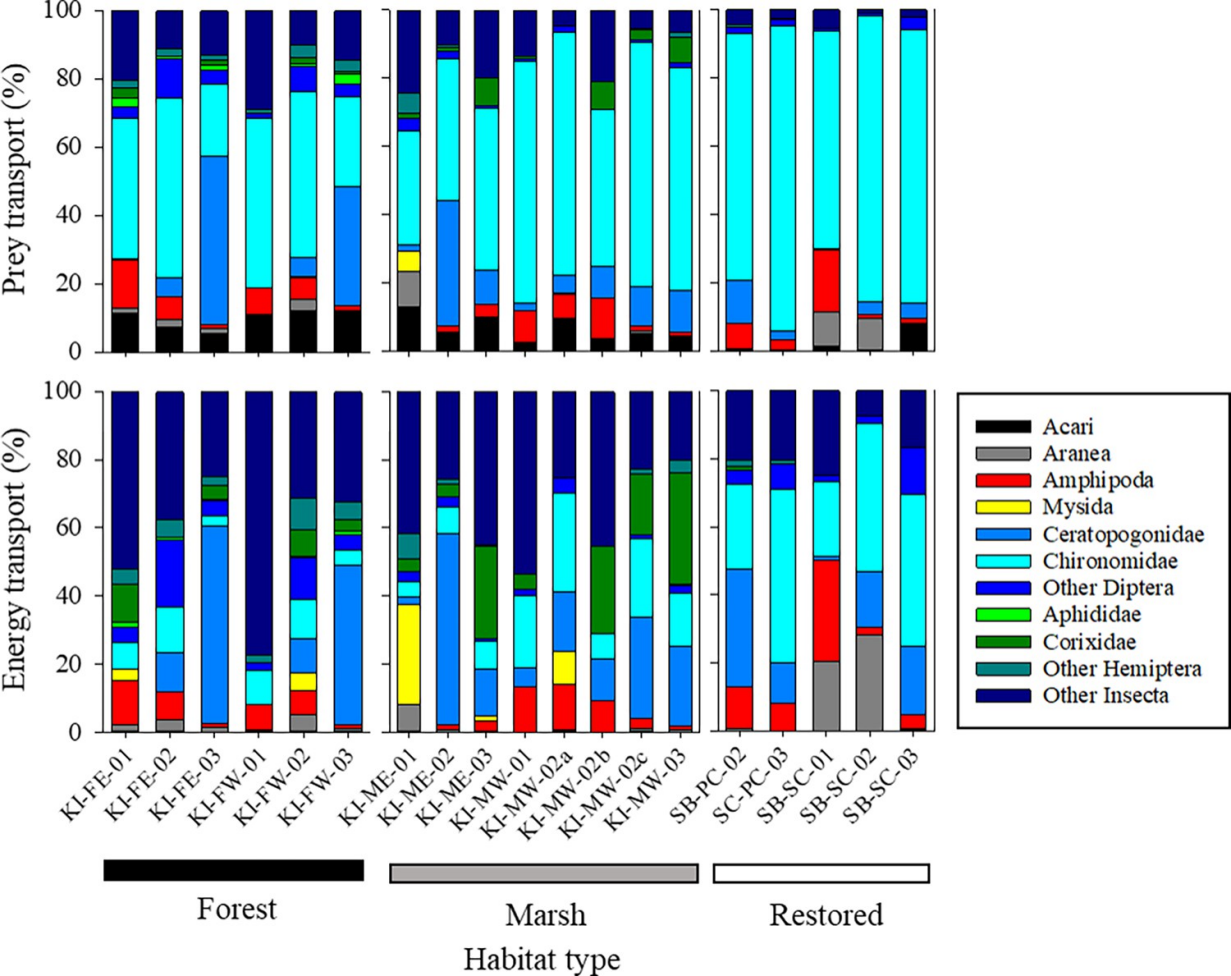
Percent of taxa contributing to prey transport (upper plots) and energy transport (lower plots) from restoration and reference tidal wetlands. Total tidal transport (prey × 10^5^) and total energy transport (kJ × 10^3^) can be found in Table 3.

Integrated (total) ebb metrics of discharge, prey transport, and prey energy transport were variable across the ebb tides we sampled (Table 3; Fig 5). Total ebb discharge across sites and dates varied from 0.14 to 5.21 ×10^4^ m^3^. For reference, the volume of an Olympic sized pool of 2 m depth is 2.5 ×10^3^ m^3^. Total prey transport measured over the sampling period ranged from 0.08 to 4.36 × 10^5^ ind/ebb tide, and the energy transport associated with the exported prey ranged from 0.05 to 2.17 × 10^3^ kJ/ebb tide (Table 3; Fig 4). Note, the percent of tide sampled should be considered in the evaluation of these values; complete ebb periods were not sampled during overbank flow, low water periods, or due to logistical constraints.

### Wetland energy subsidy

**Fig 6:**
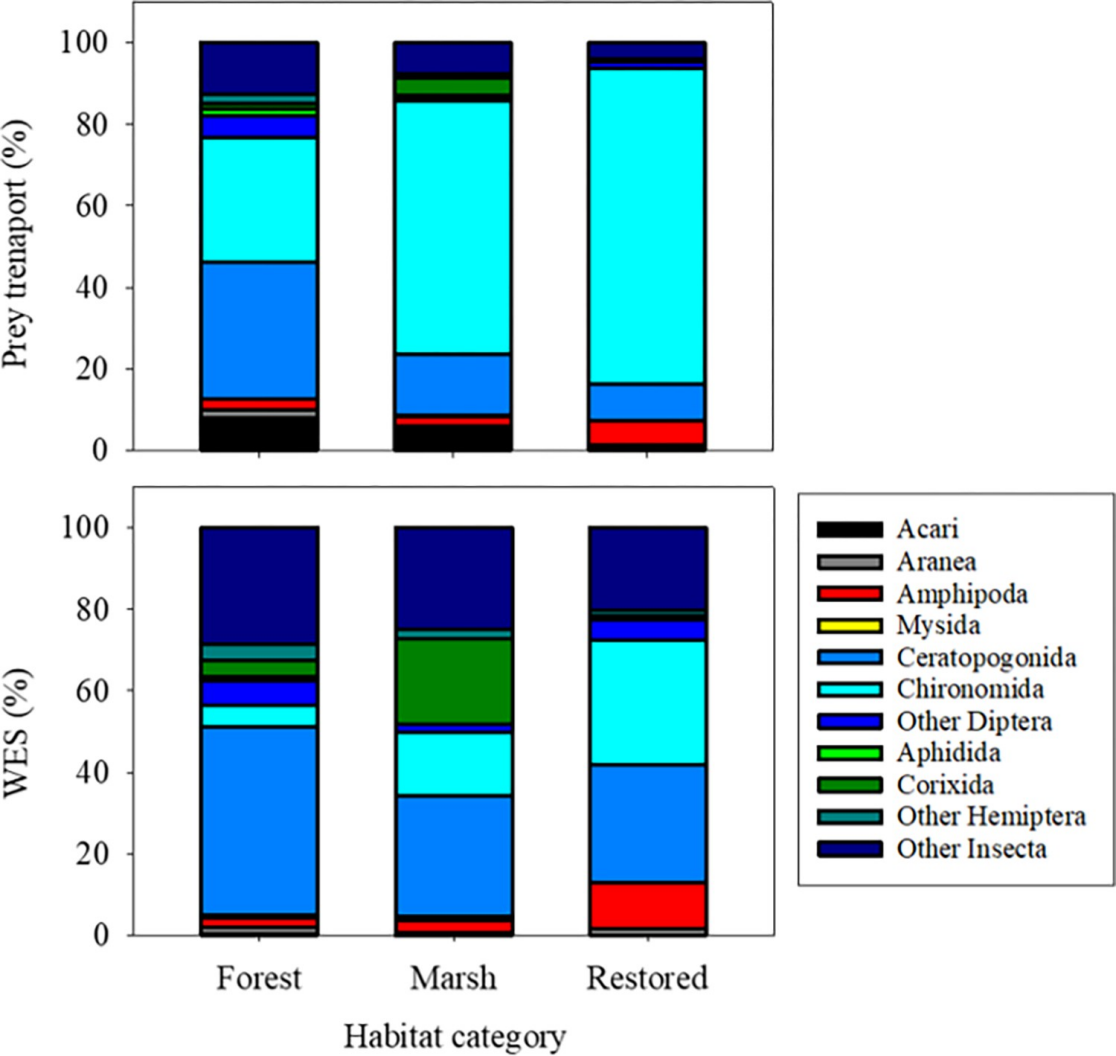
Mean percent of taxa contributing to prey transport (upper) and energy subsidy (*WES*) (lower) from reference and restored tidal wetlands in May-June 2017.

After computing the wetland energy subsidy (*WES*) from the exported energy and daily metabolic requirement of yearling-sized salmon outlined above (Table 2; Fig 2), we found mean *WES* to range between 10^1^ to >10^3^ fish/day (Table 3). Thus, tens to thousands of yearling salmon (or many more subyearlings) could have their daily energy requirements met by the rate of exported prey per individual tidal channel. We pooled observations from May and June to summarize transport and *WES* among habitats (Fig 6). The contribution of taxa to percent transport was similar at marsh and restored marsh sites (dominated by chironomids), while forested sites exported a higher proportion of Ceratopogonids. However, at all habitat types, the proportion of taxa contributing to *WES* was substantially different from that for percent prey transport (Fig 6). This is a consequence of differences in energy content between prey taxa (Fig 5). Larger, energy-rich taxa (e.g., ceratopogonids, dipterans, corixids, hemipterans, other insects) contributed more to the energy transport than smaller, energy-poor taxa (e.g., Acari, chironomids).

**Fig 7:**
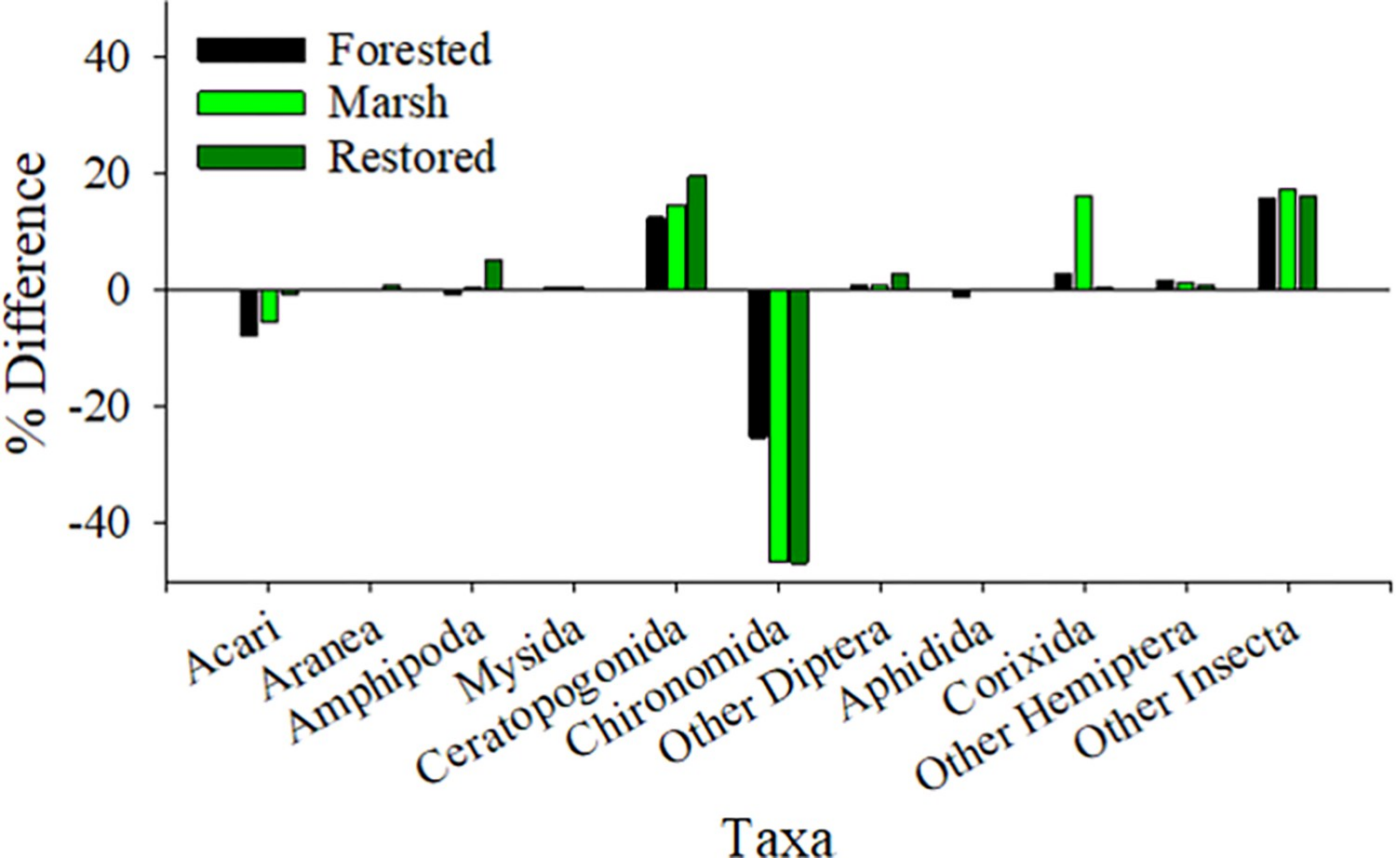
Taxa contributions to energy transport, relative to a mean proportional transport, by habitat type. Samples were pooled within the May to June 2017 period.

These findings are reflected in transport anomalies, which accentuate disproportionately high contribution to energy transport for higher energy content taxa (Fig 7). Fairly large positive energy anomalies were found in ceratopogonids and Insecta at all sites and Corixia at the marsh sites. In contrast, chironomids had very strong negative energy anomalies at all sites, indicating that although they were abundant in the material flux samples, they proportionally did not transport as much energy from the wetlands. Anomalies of the other taxa remained within ± 5%. We thus conclude variation in energy content among prey taxa can have large trophic consequences to the juvenile salmon subsidy.

## Discussion

### Prey taxa

Juvenile Pacific salmon in tidal freshwater systems are opportunistic feeders preying on a diversity of macroinvertebrate taxa, including benthic/epibenthic amphipods, ostracods, and annelids; pelagic copepods, cladocerans, and mysids; and aquatic and terrestrial insects, e.g., [35, 36, 57, 58]. Members of most of these invertebrate taxa were transported from tidal channels in our study (Fig 3). The life-cycle stage of the most dominant taxa in our samples were the aquatic larval or emerging adult phases, but aphids and members Hemiptera and Insecta included terrestrial taxa. We found dipterans (flies) and hemipterans (true bugs) of all life cycle stages comprised a high proportion of taxa transported from wetlands. Chironomid fly larvae are generally the most numerically important taxa in juvenile salmon diets [42], and were both numerically dominant and ubiquitous (frequency of occurrence >98%) in our samples. Aquatic mysids and mites and terrestrial spiders were sometimes prevalent in the net samples, but overall contributed less than 6.3% of the total numerical output. Notably, all these taxonomic groups except mysids are largely produced within wetlands [42, 59, 60]. For instance, chironomids dominated the insect drift in other wetlands studied in the LCRE [52].

In contrast, the epibenthic amphipods *Corophium salmonis* and *Americorophium spinicome* are major prey items found in salmon diets [35, 36, 42], but relatively few of these species were contained in samples from our sites. They are widespread in shallow subtidal regions outside of tidal channels. Additionally, the taxonomic composition was dominated by *Hyalella spp*., not the more commonly consumed gammarid or corophiid species. Amphipods may have low entrainment in tidal flows during daylight [61, 62], or prefer sandy substrates rather than the fine silt found in most tidal channel systems [59].

### Prey flux and transport

Few studies that measured macroinvertebrate flux or transport from TFW wetland systems are available to compare with our observations of prey fluxes ranging from 0.03 to 2.29 prey/m^2^/s and total ebb prey transports of 0.08 to 4.36 × 10^5^ prey/tide. Furthermore, we are aware of no previous study that integrated simultaneous *in situ* water velocity or discharge measurements with non-planktonic prey concentrations from TFW wetlands. Two recent studies of wetlands in the San Francisco Estuary showed the importance of vertical migration behavior and ontogeny for determining net rates transport of demersal-planktonic copepod *Pseudodiaptomus forbesi* [23, 24]. Net transport was low and generally into the wetland, leading the authors to conclude there was little subsidy for consumers outside the system [24]. Similarly, a tidal marsh in the San Francisco estuary was shown to be a sink, i.e., net import, for mature *Neomysis kadiakensis* and source, i.e, net export, for juvenile mysids [63]. This is consistent with findings for the tidal exchange between an estuary and the nearshore ocean for *Neotrypaea californiensis*, formerly known as *Callianassa californiensis* [25]. In contrast, the total annual transport of aquatic insect larvae from tidal freshwater river reaches (not wetlands) the brackish Aber Estuary (Wales, UK) was estimated to be 30.9 × 10^6^ individuals with a mass of 62.6 kg wet weight [64]. Insects dominated the export by both count and energy values. Thom et al. [65] applied a hydrodynamic model to a tidal wetland in the LCRE and demonstrated net annual export of 9.6 × 10^4^ kg particulate organic matter, especially during storm (flooding) events, to the larger ecosystem downstream. Detrital matter is a critical element of the LCRE food web [66]. Note again our study was only concerned with export, while studies over full tidal cycles are needed for net transport, e.g., [23, 25, 63, 67]. From our study and these few other investigations, we conclude transport of organic material from TFW systems can be significant.

### Wetland energy subsidy

The estimated energy content (*EC*) of invertebrate prey taxa in our study varied from 0.3 to 62.4 J/ind (Table 2). Considering the mean proportional abundance of all taxa and their corresponding energy content, we calculated an energy flux range of 0.02 to 36.06 J/m^2^/s (mean 3.55 ± 6.27 J/m^2^/s). In a lotic system (the Klamath River, California, USA), horizontal energy fluxes in the range 0.4 to 828.9 J/m^2^/s were estimated from tributary and mainstem reaches [68]. Over all sample dates, total energy transport from our TFW sites ranged from 0.05 to 2.17 × 10^3^ kJ/tide (Table 3). While total prey energy transport was positively related to discharge (see S3 Appendix), energy transport values also varied due to the energy content of specific taxa categories. The energy anomalies (Fig 6) show that, as a percentage of total transport, large and energy-rich prey taxa contributed more to the total energy transport than more numerous but lower quality items. To illustrate importance of *EC* on the prey subsidy, one can standardize taxa *EC* to that of chironomids (with an *EC* lower than all taxa except Acari, Table 2). For example, it would require seven chironomids to equal the *EC* of a single ceratopogonid, and 19.5 for a corixid. However, the concentrations of individual taxa still affected the total energy transported. For example, one can find a similar total energy transport value from a high transport of low *EC* taxa (e.g., chironomids) or a low transport of higher *EC* value (e.g., hemipterans) (see stations KI-ME-02 and SB-PC-03 in Table 2 and Fig 4 for examples). These analyses show that both prey abundance and quality (energy content) are important factors determining subsidies to the larger environment.

To evaluate how energy transport could benefit salmon, we compared the exported energy to the daily metabolic requirement of yearling Chinook Salmon (the wetland energy subsidy, *WES*). The range of *WES* values was tens to >10^3^ yearling sized fish/ebb tide. Again, these values are functions of concentration, discharge, and the energy composition of the exported prey taxa. For comparison, in small lotic systems of southeastern Alaska, macroinvertebrate concentrations were 1 to 22 prey/m^3^ and daily transport rates ranged from 5 to 6 × 10^3^ prey/d, with an energy equivalent supporting up to 2 × 10^3^ juvenile salmon per km of stream [33]. These data suggest a significant energy benefit to salmon is available from insect transport in both these studied flow regimes.

The energy density of insects is generally higher than benthic or pelagic prey in the LCRE (Fig 2), making them an optimal prey item. Chinook Salmon grew faster on dipterans in floodplain habitat than on zooplankton from mainstem river habitats [69]. Zooplankton dominated diets of juvenile salmon sampled along exposed shorelines of the mainstem Columbia River, while insects were the important prey in protected backchannel environments [70], suggesting enhanced insect availability near wetland sources. A companion study to ours found yearling Chinook Salmon and steelhead trout were feeding primarily on insects and amphipods and actively growing during rapid migration to the ocean [36]. Studies of wetland restoration sites have found insect production was enhanced and overall macroinvertebrate diversity increased following hydraulic reconnection [71], and salmon feeding in reconnected intertidal wetlands had higher insect diversity and proportional abundance than in deeper channel habitats away from wetland sources [48]. In the Sacramento River, Chinook Salmon exhibited selectivity for higher energy prey, including adult aquatic and terrestrial insects [72]. Other studies, however, show juvenile salmon select chironomids over other available and more energy rich prey [42], which may be a function of their extremely high abundance in wetland channels [59]. Patterns of insect production are clearly an important and understudied parameter for juvenile salmon feeding ecology during migration to the sea.

The basic calculations we used to estimate the energy subsidy are intended to provide a foundation for more robust modeling. To this end, several issues warrant further consideration. First, we concentrated on prey ebb export and did not sample paired flood-ebb tides to estimate net transport (input-export). Over a full semidiurnal tidal cycle, some previously exported material may be re-entrained in the next flood tide. Additionally, allochthonous material (from other sources) can be imported into a system. These processes undoubtedly occur at our sites to some degree, and not accounting for this material may overestimate export of in situ material from a given tidal creek. However, the degree of re-entrained is likely to be minimal because there would be a decreasing gradient of prey concentration emanating from the wetland mouth (source) that would dissipate with advection and mixing with mainstem water. This mixed water is not static, and during ebb reaches velocities of 0.6 to 1.0 m/s that exceed the maximum tidal creek velocity of ~0.3 m/s [73]. This indicates prey exported from a given tidal creek would be advected away from the creek mouth with little chance of re-entrainment to the source system. Only material exported near the end of the ebb tide would be in position for re-entrainment, and discharges are typically low during the end of ebb (S1 Fig in S1 Appendix). Thus, we believe an overestimation due to entrainment was possible but was unlikely to have a large effect. Short of paired flood-ebb sampling, hydrological modeling with particle tracking would help resolve this issue.

Second, the prey taxa categories we chose were based on salmon diet studies, but were necessarily broad (e.g., Diptera, Hemiptera, Insecta) and the single energy content value assigned to represent the diverse taxa comprising some of our categories resulted in high variability that increases uncertainty in the benefit to salmon (Table 2). This factor could be refined in future model iterations if *ED* and weight data were available for a wider array of taxa.

Third, we estimated the salmon daily metabolic requirement based on data from non-Chinook Salmon species and used a standard size (weight) for comparison. This standardization served to highlight a critical salmon genetic stock in the LCRE (yearling Spring Chinook), and the *WES* metric was intended to allow comparisons across sites and discharges. A more nuanced bioenergetics model incorporating salmon weight-frequency, migration timing, taxa-specific metabolic rates, and temperature data would better reflect the actual salmon migration patterns and corresponding environmental conditions occurring at the varying hydrogeomorphic reaches. Note for example that *WES* is linearly related to metabolic rate in our static model but requires a power function when considering a wider temperature range. Such a model could investigate the importance of wetland subsidies to other salmon populations in a more holistic matter, e.g., [74].

Finally, our prey samples were collected during within-channel flow, which does not account for the effect of large discharges during overbank flow. Overbank flow in TFW wetlands occurs regularly with the spring-neap cycle and high river stage, especially at lower elevation emergent marsh sites. Prey ebb transport was thus underestimated when we could not sample the full ebb discharge (see percent tide sampled in Table 3). A hydrological model at the wetland scale could enable fuller accounting over synodic and seasonal time scales, e.g., [67, 75, 76], including estimation of net transport rates, e.g. [24].

### General site comparisons

Although lack of sufficient replication at the site scale prevents rigorous site comparisons, we were able to use replicate sample dates (May and June) to explore basic trends among sites. We found variation in the composition of transported prey across sample dates for the three tidal freshwater habitat types we studied (Fig 5). In general, chironomids dominated export from the marsh sites while roughly equal proportions of chironomids and ceratopogonids were exported from the forested sites (Figs 5 and 6). There was significantly higher diversity prey from Sitka spruce forested sites than from emergent marshes, but we observed no practical difference in number of taxa categories (see S2 Appendix). Insect and arachnid diversity from fall-out traps prey have been found to vary by site and also over seasonal time scales [59], but we are unaware of other documented relationships between habitat type (vegetation) and prey production in TFW settings. The broad taxonomic categories used in our study likely masked a true measure of habitat-specific diversity. We additionally tested for the effects of stage of tide and bankfull area on key metrics (*C*_*T*_, *U*, *Q*, *F*_*I*_, and *T*_*I*_) among habitat types, finding these two variables were poor predictors, except for discharge which was positively correlated with bankfull area (see S3 Appendix). Larger wetland watersheds generally have wider channel mouth cross-sectional area [16], resulting in higher discharges. Regardless of vegetation, size, and discharge, and limitations on statistical comparisons, all three wetland habitat types exported significant amounts of macroinvertebrate prey and energy to the mainstem LCRE.

### Sources of invertebrate flux

The availability of insect prey for fish is affected by both physical and behavioral factors [62, 77, 78]. Physical factors include direct fall-out, entrainment during high water (tidal stage or flood events), and scour from benthic habitats [79]. Behavioral factors include emergence (life-cycle functions) or active drift (habitat/position change) [24, 30, 32, 62]. Based on numbers of terrestrial organisms caught in fall-out traps, aquatic organisms metamorphosing into the adult phase that are caught in emergence traps, and organisms captured in benthic cores, the inputs of insects into tidal channels can be prodigious. In the LCRE, total insect vertical fluxes in May and June ranged from 405 to 1,095 prey/m^2^/d at emergent marsh sites, compared with ~200 prey/m^2^/d from a Karlson Island forested site, as estimated from [59]. From a variety of U.S. West Coast estuaries, variable rates of mean terrestrial arthropod inputs have been measured (mean 37.2 ± 60.7 prey/m^2^/hr) [38]. Emergence rates (all insect taxa) in LCRE wetland tidal channels were up to 140 prey/m^2^/d, while chironomids peaked at about 40 prey/m^2^/d [60]. These data indicate high numbers of aquatic and terrestrial insects moving through TFW channels, similar to those found in our ebb tide transport samples.

In riverine systems, vertical arthropod fluxes, which represent terrestrial subsidies to aquatic systems, contribute significantly to stream ecosystem food webs. For example, in southeastern Alaska (USA), vertical fluxes of terrestrial invertebrates (mostly dipterans) were found to average 66 mg/m^2^/d [80], while fluxes (mostly terrestrial insects) into streams averaged 80 mg/m^2^/d [81]. Mean vertical fluxes up to 130 mg/m^2^/d from deciduous forest canopies were measured during the growing season, with fluxes falling to low values during winter [79]. Many of the lotic studies demonstrate invertebrate subsidies enhance stream salmonid diets [30, 80, 82]. As a comparison, with an average energy density for insects of 8.92 J/mg, energy fluxes for these Alaska sites range from 0.59 to 1.16 kJ/m^2^/d. Thus, in both tidal freshwater and lotic environments, salmonids characteristically feed on arthropods (primarily insects) of terrestrial as well as aquatic origin and subsidies from allochthonous sources can be significant.

While information from TFW sites is limited, various lotic studies have demonstrated that vegetation type affects terrestrial prey input to streams. For instance, riparian zones dominated by red alder (*Acer* spp.) had higher vertical fluxes than those with coniferous cover (including Sitka spruce found in our forested sites) [81]. Tree density was also found to be positively related to prey subsidies [31]. Fall-out from trees may rely on meteorological events (wind, rain) not captured in our sampling design. Further studies are needed in TFW sites to better quantify differences in taxon-specific energy content that may occur between forested and emergent marsh habitats that could have an episodic vertical flux.

### Tidal channel mouths as feeding hotspots

In lotic systems, researchers have conceptualized the resource subsidy concept, whereby primary and secondary production originating upstream over a wide area of varied sites, e.g., terrestrial and riparian zones, is packaged into arthropod prey that become concentrated in narrow pathways such as a riverine riffle above a pool [30, 34, 83]. Other studies have shown higher fish concentrations at river tributary junctions than in upstream reaches [84, 85], and in saltmarshes some predators concentrate at channel mouths presumably to feed [86]. Concentrations of prey at predictable downstream locations has been viewed as an upstream / terrestrial subsidy to downstream predators [85]. Analogously, the mouths of TFW wetland channels are sites of enhanced prey transport concentrated from the larger wetland complex of channels, vegetated marsh surface, and riparian cover (Fig 1). Channel mouths thus constitute the interface between disparate sites of prey production and their delivery to the deeper subtidal river environment. This enhancement occurs over a narrow spatial zone (generally <10^2^ m) and at a predicable (tidal) time scale. Predators, and particularly larger salmon migrants that do not typically enter shallow intertidal channels, can still benefit from wetland production by congregating during ebb tide flows around the mouths of TFW tidal channels. This hypothesis has not been investigated in the LCRE, but has implications for wetland restoration strategies, for example, optimizing the number, dimensions, and orientations of excavated channels in wetland reconnection projects [16, 87].

### Implications

Since the late 1800s, almost three-quarters of the total historical area of vegetated tidal wetlands of the LCRE have been lost to diking, filling, and bank hardening, combined with flow regulation and other modifications [37, 38, 88]. In response, a broad campaign has been initiated to restore connectivity between degraded LCRE wetlands and the mainstem river [39, 41]. Many of these restoration projects are explicitly designed to improve access for juvenile Pacific salmon to productive wetland interiors [40, 89], and restoration has indeed increased hydrological connections and opportunities for juvenile salmon at many locations in the LCRE [39, 41, 90]. Our study of prey flux and transport rates, though, shows ecological benefits of restoring wetlands exceed their physical boundaries. Both forested and emergent wetlands, as well as restoring marshes of differing ecological trajectories, facilitate export of prey resources to juvenile salmon in deeper water environments outside wetlands, including the mainstem river [36]. Our wetland energy subsidy modeling, targeting larger and endangered salmon stocks, indicates that thousands of juvenile salmon per day are supported from the few tidal channels we studied, and hundreds more tidal channels exist in our study reach alone. Intuitively, scaling up the cumulative transport rate to a hydrogeomorphic reach scale suggests these wetland complexes likely provide a vast resource of prey for most or all salmon species and stocks during migration to the ocean. Previous assessments of benefits to salmon from proposed restoration projects focused mostly on the expected direct, onsite benefits derived from foraging and refuge from predators of fish within the wetlands [91]. Our subsidy measurements provide evidence that the effective ecological footprint of a given wetland can greatly exceed its physical area. The cumulative effect of subsidies from wetland complexes likely provides a substantial benefit for salmon and other organisms, and increases the overall resilience of populations to environmental alteration.

## Supporting information

S1 Appendix Example of time series data used for transport calculations. (PDF)

S2 Appendix Site comparisons. (PDF)

S3 Appendix Regression analyses. (PDF)

## References

[1] Dame RF, Childers D, Koepfler E. A geohydrologic continuum theory for the spatial and temporal evolution of marsh-estuarine ecosystems. Neth J Sea Res. 1992;30:63–72.

[2] Childers DL, Day JW, McKellar HN. Twenty more years of marsh and estuarine flux studies: revisiting Nixon (1980). In Weinstein MP, Kreeger DA (Editors). Concepts and Controversies in Tidal Marsh Ecology. Kluwer Academic Publishers, 2000.

[3] Land M, Granéli W, Grimvall A, Hoffmann CC, Mitsch WJ, Tonderski KS, et al. How effective are created or restored freshwater wetlands for nitrogen and phosphorus removal? A systematic review. Environ Evid. 2016;5:9. doi: 10.1186/s13750-016-0060-0

[4] Zedler JB, Kercher S. Wetland resources: status, trends, ecosystem services, and restorability. Annu Rev Environ Resour. 2005;30:39–74.

[5] Krauss KW, Noe GB, Duberstein JA, Conner WH, Stagg CL, Cormier N, et al. The role of the upper tidal estuary in wetland blue carbon storage and flux. Global Biogeochem Cycles. 2018;32:817–839. doi: 10.1029/2018GB005897

[6] Rolls RJ, Leigh C, Sheldon F. Mechanistic effects of low-flow hydrology on riverine ecosystems: ecological principles and consequences of alteration. Freshw Sci. 2012;31(4):1163–1186.

[7] Magnusson A, Hilborn R. Estuarine influence on survival rates of Coho (*Oncorhynchus kisutch*) and Chinook salmon (*Oncorhynchus tshawytscha*) released from hatcheries on the US Pacific Coast. Estuaries. 2003;26:1094–1103.

[8] Odum EP. A research challenge: evaluating the productivity of coastal and estuarine water. Proceedings of the 2nd Sea Grant Conference, University of Rhode Island, Kingston, 1968. pp. 63–64.

[9] Dame RF, Allen DM. Between estuaries and the sea. J Exp Mar Biol Ecol. 1996;200:169–185.

[10] Weinstein MP, Litvin SY, Krebs JM. Restoration ecology: Ecological fidelity, restoration metrics, and a systems perspective. Ecol Eng. 2014;65:71–87.

[11] Hume TM, Bell RG. Methods for determining tidal flows and material fluxes in estuarine cross-sections. Water Quality Centre Publ. 1993;22. National Institute of Water and Atmospheric Research, Hamilton, New Zealand.

[12] Nixon SW. 1980. Between coastal marshes and coastal waters: A review of twenty years of speculation and research on the role of salt marshes in estuarine productivity and water chemistry. In P. Hamilton and K. B MacDonald, editors. Estuarine and wetland processes. Plenum Press, New York; 1980. pp. 437–525.

[13] Fagherazzi S, Wiberg PL, Temmerman S, Struyf E, Zhao Y, Raymond PA. Fluxes of water, sediments, and biogeochemical compounds in salt marshes. Ecol Process. 2013;2:3. doi: 10.1186/2192-1709-2-3

[14] Santos IR, Burdige DJ, Jennerjahn TC, Bouillon S, Cabral A, Serrano O, et al. The renaissance of Odum’s outwelling hypothesis in ’Blue Carbon’ science. Estuar Coast Shelf Sci. 2021;255: doi: 10.1016/j.ecss.2021.107361

[15] Borde AB, Diefenderfer HL, Cullinan VI, Zimmerman SA, Thom RM. Ecohydrology of wetland plant communities along an estuarine to tidal river gradient. Ecosphere. 2020;11(9):e03185. doi: 10.1002/ecs2.3185

[16] Diefenderfer HL, Borde AB, Cullinan VI. Floodplain wetland channel planform, cross-sectional morphology, and sediment characteristics along an estuarine to tidal river gradient. Journal of Geophysical Research: Earth Surface. 2021;126, e2019JF005391. doi: 10.1029/2019JF005391

[17] Gladstone-Gallagher RV, Sandwell DR, Lohrer AM, Lundquist CJ, Pilditch CA. Quantifying macrodetritus fluxes from a small temperate estuary. Mar Freshw Res. 2017;68:2289–2305. doi: 10.1071/MF16408

[18] Clark JB, Long W, Hood RR. A comprehensive estuarine dissolved organic carbon budget using an enhanced biogeochemical model. J Geophys Res Biogeosci. 2020. e2019JG005442.

[19] Voulgaris G, Meyers ST. Temporal variability of hydrodynamics, sediment concentration and sediment settling velocity in a tidal creek. Cont Shelf Res. 2004;24:1659–1683.

[20] Ganju NK, Nidzieko NJ, Kirwan ML. Inferring tidal wetland stability from channel sediment fluxes: Observations and a conceptual model. J Geophys Res Earth Surf. 2013;118:2045–2058. doi: 10.1002/jgrf.20143

[21] Carlson DM. The ecological role of zooplankton in a Long Island salt marsh. Estuaries. 1978;1:85–92.

[22] Roegner GC. Hydrodynamic control of the supply of suspended chlorophyll a to estuarine infaunal bivalves. Estuar Coast Shelf Sci. 1998;47:369–384.

[23] Kimmerer W, Ignoffo TR, Bemowski B, Modéran J, Holmes A, Bergamaschi B. Zooplankton dynamics in the Cache Slough complex of the upper San Francisco Estuary. San Francisco Estuary and Watershed Science. 2018;16(3). doi: 10.15447/sfews.2018v16iss3art4

[24] Yelton R, Slaughter AM, Kimmerer WJ. Diel behaviors of zooplankton interact with tidal patterns to drive spatial subsidies in the Northern San Francisco Estuary. Estuar Coasts. 2022;45:1728–1748. doi: 10.1007/s12237-021-01036-8

[25] Johnson GE, Gonor JJ. The tidal exchange of *Callianassa californiensis* (Crustacea, Decapoda) larvae between the ocean and the Salmon River estuary, Oregon. Estuar Coast Shelf Sci. 1982;14:501–514.

[26] Lago RP. Tidal exchange of larvae of *Sesarma catenata* (Decapoda, Brachyura) in the Swartkops estuary, South Africa. South African Journal of Zoology. 1993;28:182–191.

[27] Roegner GC. Transport of larval molluscs through a shallow estuary. J Plankton Res. 2000;22:1779–1800.

[28] Kneib R. The role of tidal marshes in the ecology of estuarine nekton. In Ansell AD, Gibson RN, Barnes, editors. Oceanography and Marine Biology: an Annual Review. London: UCL Press; 1997;35:163–220.

[29] Craig JK, Crowder LB. Factors influencing habitat selection in fishes with a review of marsh ecosystems. In Concepts and controversies in tidal marsh ecology. Edited by Weinstein MP, Kreeger DA. Kluwer Academic Publishers, Dordrecht, Netherlands. pp. 241–266. 2000.

[30] Nakano S, Miyasaka H, Kuhara N. Terrestrial-aquatic linkages: riparian arthropod inputs alter trophic cascades in a stream food web. Ecology. 1999;80:2435–2441.

[31] Wipfli MS, Musslewhite J. Density of red alder (*Alnus rubra*) in headwaters influences invertebrate and detritus subsidies to downstream fish habitats in Alaska. Hydrobiologia. 2004;520:153–163.

[32] Lancaster J, Hildrew AG, Gjerlov C. Invertebrate drift and longitudinal transport processes in streams. Can J Fish Aquat Sci. 1996;53:572–582. doi: 10.1139/f95-217

[33] Wipfli MS, Gregovich DP. Export of invertebrates and detritus from fishless headwater streams in southeastern Alaska: implications for downstream salmonid production. Freshw Biol. 2002;47:957–969.

[34] Wipfli MS, Richardson JS, Naiman RJ. Ecological linkages between headwaters and downstream ecosystems: Transport of organic matter, invertebrates, and wood down headwater channels. J Amer Water Res Assoc. 2007;43:72–85.

[35] Bottom DL, Jones KK. Species composition, distribution, and invertebrate prey of fish assemblages in the Columbia River Estuary. Prog Oceanogr. 1990;25:243–270.

[36] Weitkamp LA, Beckman BR, Van Doornik D, Munguia A, Hunsicker M, Journey M. Life in the fast lane: feeding and growth of juvenile steelhead and Chinook Salmon in mainstem habitats of the Columbia River Estuary. Trans Am Fish Soc. 2022; doi: 10.1002/tafs.10376

[37] Marcoe K, Pilson S. Habitat change in the lower Columbia River estuary, 1870–2009. J Coast Conserv. 2017;21:505–525. doi: 10.1007/s11852-017-0523-7

[38] Brophy LS, Greene CM, Hare VC, Holycross B, Lanier A, Heady WN, et al. Insights into estuary habitat loss in the Western United States using a new method for mapping maximum extent of tidal wetlands. PLoS ONE. 2019;14(8): e0218558. doi: 10.1371/journal.pone.0218558 31412030 PMC6693690

[39] Ebberts BD, Zelinsky BD, Karnezis JP, Studebaker CA, Lopez-Johnston S, Creason AM, et al. 2017. Implementing and institutionalizing adaptive management of the Columbia Estuary Ecosystem Restoration Program. Restor Ecol. 2017. doi: 10.1111/rec.12562

[40] Krueger KL, Bottom DL, Hood WG, Johnson GE, Jones KK, Thom RM. An expert panel process to evaluate habitat restoration actions in the Columbia River estuary. J Environ Manage. 2017;188:337–350. doi: 10.1016/j.jenvman.2016.11.028 28006743

[41] Littles C, Karnezis J, Blauvelt K, Creason A, Diefenderfer H, Johnson G, et al. Adaptive management of large-scale ecosystem restoration: increasing certainty of habitat outcomes in the Columbia River Estuary, USA. Restor Ecol. 2022; doi: 10.1111/rec.13634

[42] Bottom DL, Baptista A, Burke J, Campbell L, Casillas, Craig B, et al. Estuarine habitat and juvenile salmon: current and historical linkages in the lower Columbia River and estuary: Final report 2002–08. Report of Research by Fish Ecology Division, NOAA Fisheries to Portland District, U.S. Army Corps of Engineers. 2011. Available from: https://apps.dtic.mil/sti/pdfs/ADA581333.pdf.

[43] Roegner GC, Weitkamp LA, Teel D. Comparative use by Pacific salmon of shallow and deep water habitats in the Columbia River estuary prior to ocean entry. Mar Coast Fisheries: Dynamics, Management, and Ecosystem Science. 2016;8:536–553. doi: 10.1080/19425120.2016.1227889

[44] SonTek/YSI Inc. 2017. SonTek-IQ Series Intelligent Flow Featuring SmartPulseHD User’s Manual. Tech. Report. Xylem, San Diego, CA.

[45] Harding SF, Coleman AM, Roegner GC. 2020. Comparison of experimental and computational methods for discharge measurements from tidal wetlands. River Res Appl. 2020;36:1954–1961.

[46] David AT, Goertler PAL, Munsch SH, Jones BR, Simenstad CA, Toft JD, et al. Influences of natural and anthropogenic factors and tidal restoration on terrestrial arthropod assemblages in West Coast North American estuarine wetlands. Estuaries Coast. 2016; doi: 10.1007/s12237-016-0091-3

[47] Gray A. The Salmon River estuary: restoring tidal inundation and tracking ecosystem response. Doctoral dissertation. University of Washington. 2005. Available from: https://www.researchgate.net/profile/Ayesha-Gray/publication/33515883_The_Salmon_River_Estuary_Restoring_Tidal_Inundation_and_Tracking_Ecosystem_Response/links/00b49527980d29669a000000/The-Salmon-River-Estuary-Restoring-Tidal-Inundation-and-Tracking-Ecosystem-Response.pdf.

[48] Roegner GC, Dawley EM, Russell M, Whiting A, Teel DJ. Juvenile salmonid use of reconnected tidal freshwater wetlands in Grays River, lower Columbia River basin. Trans Am. Fish Soc. 2010;139:1211–1232.

[49] Chabot D, McKenzie DJ, Craig JF. Metabolic rate in fishes: definitions, methods and significance for conservation physiology. J Fish Bio. 2016;88:1–9. doi: 10.1111/jfb.12873 26768969

[50] Brett JR. Scope for metabolism and growth of sockeye salmon, *Oncorhynchus nerka*, and some related energetics. J Fish Res Bd Can. 1976;33:307–313.

[51] Kaushik SJ, Medale F. Energy requirements, utilization and dietary supply to salmonids. Aquaculture. 1994;124:81–97.

[52] Roegner GC, Teel DJ. Density and condition of subyearling Chinook salmon in the lower Columbia River and estuary in relation to water temperature and genetic stock of origin. Trans Am. Fish Soc. 2014;143: 1161–1176. doi: 10.1080/00028487.2014.918055

[53] Kaushik SJ, Gomes EF. Effect of frequency of feeding on nitrogen and energy balance in rainbow trout under maintenance conditions. Aquaculture. 1988;73:207–216.

[54] Storebakken T, Hung SSO, Calvert CC, Plisetskaya EM. Nutrient partitioning in rainbow trout at different feeding rates. Aquaculture. 1991;96:191–203.

[55] Cho CY, Bureau DP. Determination of the energy requirements of fish with particular reference to salmonids. J Appl Ichthyol. 1995;11:141–163.

[56] Millidine KJ, Armstrong JD, Metcalfe NB. Juvenile salmon with high standard metabolic rates have higher energy costs but can process meals faster. Proc Biol Sci. 2009;276:2103–2108. doi: 10.1098/rspb.2009.0080 19324750 PMC2677234

[57] Levings CD, Boyle DE, Whitehouse TR. Distribution and feeding of juvenile Pacific salmon in freshwater tidal channels of the lower Fraser River, British Columbia. Fish Manag Ecol. 1995;2:299–308.

[58] Heerhartz SM, Toft JD. Movement patterns and feeding behavior of juvenile salmon (*Oncorhynchus spp*.) along armored and unarmored estuarine shorelines. Environ Biol Fishes. 2015;98:1501–1511. doi: 10.1007/s10641-015-0377-5

[59] Lott MA. Habitat-specific feeding ecology of ocean-type juvenile Chinook salmon in the lower Columbia River estuary. M.Sc. Thesis, University of Washington. 2004. Available from: http://citeseerx.ist.psu.edu/viewdoc/download?doi=10.1.1.596.8104&rep=rep1&type=pdf.

[60] Ramirez MF. Emergent aquatic insects: assemblage structure and patterns of availability in freshwater wetlands of the lower Columbia River Estuary. M.Sc. Thesis, University of Washington. 2008.

[61] Davis JS. Diel activity of benthic crustaceans in the Columbia River estuary. M.Sc. Thesis, Oregon State University. 1978. Available from: https://ir.library.oregonstate.edu/downloads/5d86p440w.

[62] Naman SM, Rosenfeld JS, Richardson JS. Causes and consequences of invertebrate drift in running waters: from individuals to populations and trophic fluxes. Can J Fish Aquat Sci. 2016;73:1292–1305. doi: 10.1139/cjfas-2015-0363

[63] Dean AF, Bollens SM, Simenstad CA, Cordell J. Marshes as sources or sinks of an estuarine mysid: demographic patterns and tidal flux of *Neomysis kadiakensis* at China Camp marsh, San Francisco estuary. Estuar Coast Mar Sci. 2005;63:1–11.

[64] Williams DD, Williams NE. Seasonal variation, export dynamics and consumption of freshwater invertebrates in an estuarine environment. Estuar Coast Shelf Sci. 1998;46:393–410.

[65] Thom R, Breithaupt S, Diefenderfer H, Borde A, Roegner G, Johnson G, et al. Storm-driven particulate organic matter flux connects a tidal tributary floodplain wetland, mainstem river, and estuary. Ecol Appl. 2018;28:1420–1434. doi: 10.1002/eap.1759 30035832

[66] Maier GO, Simenstad CA. The role of marsh-derived macrodetritus to the food webs of juvenile Chinook salmon in a large altered estuary. Estuar Coasts. 2009;32:984–998. doi: 10.1007/s12237-009-9197-1

[67] Macdonald JS, Kistritz RU, Farrell M. An examination of the effects of slough habitat reclamation in the lower Fraser River, British Columbia: detrital and invertebrate flux, rearing and diets of juvenile salmon. Canadian Technical Report of Fisheries and Aquatic Science. 1990;1731, 68 pp.

[68] Brewitt KS, Danner EM, Moore JW. Hot eats and cool channels: juvenile Pacific salmonids use mainstem prey while in thermal refuges. Can J Fish Aquat Sci. 2017;74:1588–1602.

[69] Sommer TR, Nobriga ML, Harrell WC, Batham W, Kimmerer WJ. Floodplain rearing of juvenile chinook salmon: evidence of enhanced growth and survival. Can J Fish Aquat Sci. 2001;58:325–333.

[70] Goertler PAL, Simenstad CA, Bottom DL, Hinton S, Stamatiou L. Estuarine habitat and demographic factors affect juvenile Chinook (*Oncorhynchus tshawytscha*) growth variability in a large freshwater tidal estuary. Estuaries Coast. 2016;39:542–559.

[71] Haskell CA, Tiffan KF. Crims Island—Restoration and monitoring of juvenile salmon rearing habitat in the Columbia River Estuary, Oregon, 2004–10: U.S. Geological Survey Scientific Investigations Report 2011–5022, 2011. Available from: https://pubs.usgs.gov/sir/2011/5022/.

[72] Goertler P, Jones K, Cordell J, Schreier B, Sommer T. Effects of extreme hydrologic regimes on juvenile Chinook Salmon prey resources and diet composition in a large river floodplain. Trans Am Fish Soc. 2018;147:287–299. doi: 10.1002/tafs.10028

[73] Sandbach SD, Nicholas AP, Ashworth PJ, Best JL, Keevil CE, Parsons DR, et al. Hydrodynamic modelling of tidal-fluvial flows in a large river estuary. Estuar Coast Shelf Sci. 2018;212:176–188.

[74] Roegner GC, Johnson GE, Coleman AM. Indexing habitat opportunity for juvenile anadromous fishes in tidal-fluvial wetland systems. Ecol Indic. 2021;124:107422. doi: 10.1016/j.ecolind.2021.107422

[75] Diefenderfer HL, Coleman AM, Borde AB, Sinks IA. Hydraulic geometry and microtopography of tidal freshwater forested wetlands and implications for restoration, Columbia River, U.S.A. Ecohydrology and Hydrobiology. 2008;8: 339–361. doi: 10.2478/V10104-009-0027-7

[76] Coleman AM, Diefenderfer HL, Ward DL, Borde AB. A spatially based area-time inundation index model developed to assess habitat opportunity in tidal-fluvial wetlands and restoration sites. Ecol Eng. 2015;82: 624–642. doi: 10.1016/j.ecoleng.2015.05.006

[77] Poff NL, DeCino RD, Ward JV. Size-dependent drift responses of mayflies to experimental hydrologic variation: active predator avoidance or passive hydrodynamic displacement? Oecologia 1991;88:577–586. doi: 10.1007/BF00317723 28312630

[78] Douglas PL, Forrester GE, Cooper SD. Effects of trout on the diel periodicity of drifting in baetid mayflies. Oecologia 1994;98:48–56. doi: 10.1007/BF00326089 28312795

[79] Nakano S, Murakami M. Reciprocal subsidies: dynamic interdependence between terrestrial and aquatic food webs. Proc Natl Acad Sci U S A. 2001;98:166–170. doi: 10.1073/pnas.98.1.166 11136253 PMC14562

[80] Wipfli MS. Terrestrial invertebrates as salmonid prey and nitrogen sources in streams: contrasting old-growth and young-growth riparian forests in southeastern Alaska, U.S.A. Can J Fish Aquat Sci. 1997;54:1259–1269.

[81] Allan JD, Wipfli MS, Caouette JP, Prussian A, Rodgers J. Influence of streamside vegetation on inputs of terrestrial invertebrates to salmon food webs. Can J Fish Aquat Sci. 2003;60:309–320.

[82] Weber N, Bouwes N, Jordan CE. Estimation of salmonid habitat growth potential through measurements of invertebrate food abundance and temperature. Can J Fish Aquat Sci. 2014;71:1158–1170.

[83] Wipfli MS, Baxter CV. Linking ecosystems, food webs, and fish production: subsidies in salmonid watersheds. Fisheries. 2010;35:373–387.

[84] Benda LE, Poff NL, Miller D, Dunne T, Reeves G, Pess G, et al. The network dynamics hypothesis: how channel networks structure riverine habitats. BioScience. 2004;54(5):413–427.

[85] Kiffney PM, Greene C, Hall J, Davies J. Gradients in habitat heterogeneity, productivity, and biodiversity at tributary junctions. Can J Fish Aquat Sci. 2006;63:2518–2530.

[86] Tupper M, Able KW. Movements and food habits of striped bass (*Morone saxatilis*) in Delaware Bay (USA) salt marshes: comparison of a restored and a reference marsh. Mar Biol. 2000;137:1049–1058.

[87] Hood WG. Scaling tidal channel geometry with marsh island area: a tool for habitat restoration, linked to channel formation process. Water Resour Res. 2007;43, W03409, doi: 10.1029/2006WR005083

[88] Kukulka T, Jay DA. Impacts of Columbia River discharge on salmonid habitat: 2. Changes in shallow-water habitat. J Geophys Res. 2003,108: 3294. doi: 10.1029/2003JC001829, C9.

[89] Simenstad CA, Cordell JR. Ecological assessment criteria for restoring anadromous salmonid habitat in Pacific Northwest estuaries. Ecol Eng. 2000;15, 283e302. doi: 10.1016/S0925-8574(00)00082-3

[90] Diefenderfer HL, Johnson GE, Thom RM, Buenau KE, Weitkamp LA, Woodley CM, et al. Evidence-based Evaluation of the Cumulative Effects of Ecosystem Restoration. Ecosphere. 2016;7(3):e01242. doi: 10.1002/ecs2.1242

[91] David AT, Ellings CS, Woo I, Simenstad CA, Takekawa JY, Turner KL, et al. Foraging and growth potential of juvenile Chinook salmon after tidal restoration of a large river delta. Trans Am Fish Soc. 2014;143: 1515–1529. doi: 10.1080/00028487.2014.945663

