# Supplementary material for: Export of macroinvertebrate prey from tidal freshwater wetlands provides a significant energy subsidy for outmigrating juvenile salmon
Source: PLoS One. 2023 Mar 17;18(3):e0282655. doi: 10.1371/journal.pone.0282655 (PMC10022792; doi:10.1371/journal.pone.0282655)
Supplement: S1 Appendix — (PDF) [file pone.0282655.s001.pdf]

S1 Appendix. Example transport calculation

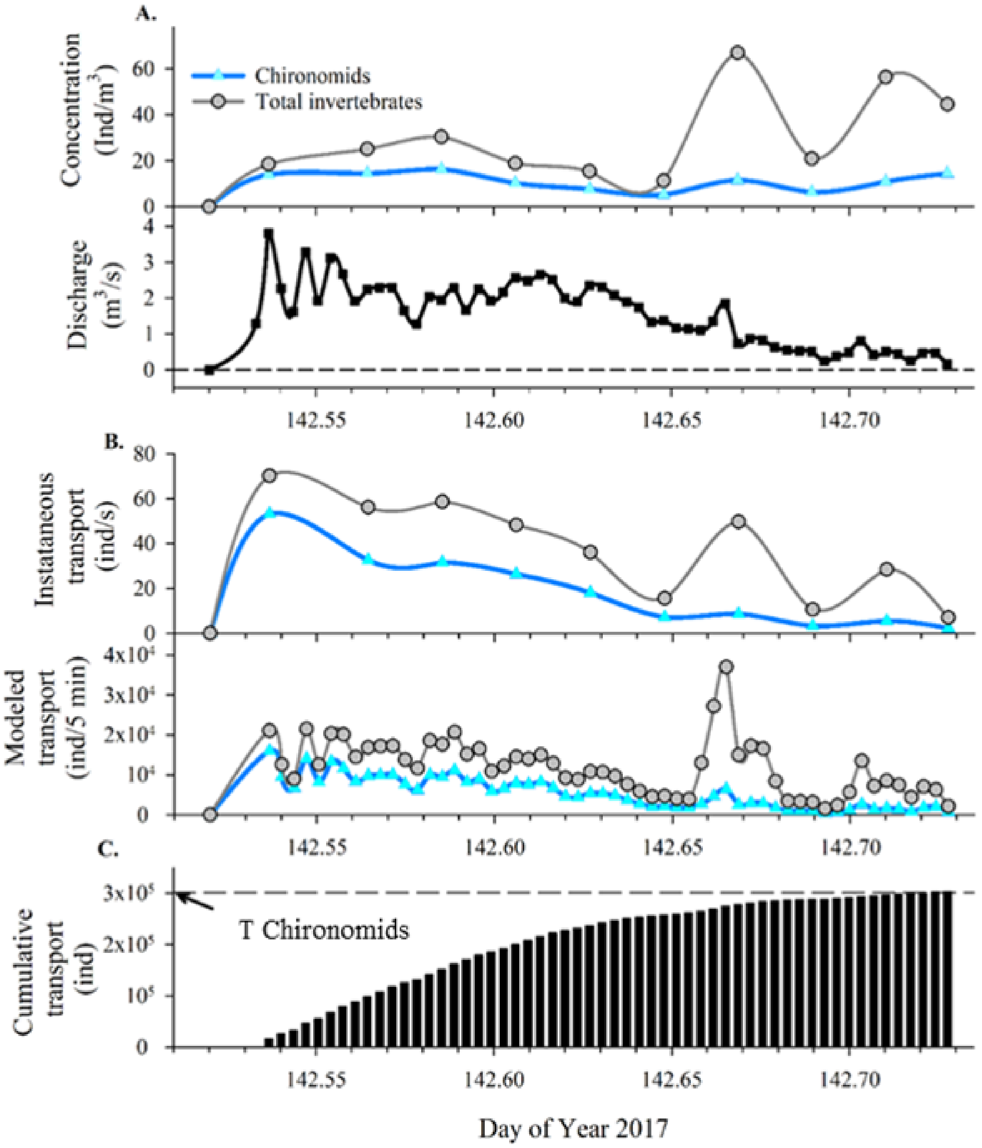

S1 Fig. Example of time series data used for transport calculations. A. Field measurements. Upper panel, discharge; Lower panel, chironomid and total invertebrate concentrations. B. Calculated transports. Upper panel, instantaneous transports; lower panel, integrated transports. C. Cumulative transport of chironomids, and resultant total ebb transport (T). Data from Steamboat primary channel (SB-PC-01) collected on 22 May 2017.
