# Supplementary material for: Export of macroinvertebrate prey from tidal freshwater wetlands provides a significant energy subsidy for outmigrating juvenile salmon
Source: PLoS One. 2023 Mar 17;18(3):e0282655. doi: 10.1371/journal.pone.0282655 (PMC10022792; doi:10.1371/journal.pone.0282655)
Supplement: S2 Appendix — (PDF) [file pone.0282655.s002.pdf]

S2 Appendix. Site comparisons

We compared mean concentration, diversity (H'), and number of taxa (ST) by habitat type using single factor ANOVA and SNK post-hoc tests (S2 Fig). Mean concentration was highest at the marsh site, but not significantly so due to high variation, while concentrations at the forested sites were significantly lower than the restored marsh sites. Mean diversity was highest at the forested site and lowest at the restored site. The mean number of taxa was lower at the restored than at the reference sites, but the mean difference was slight.

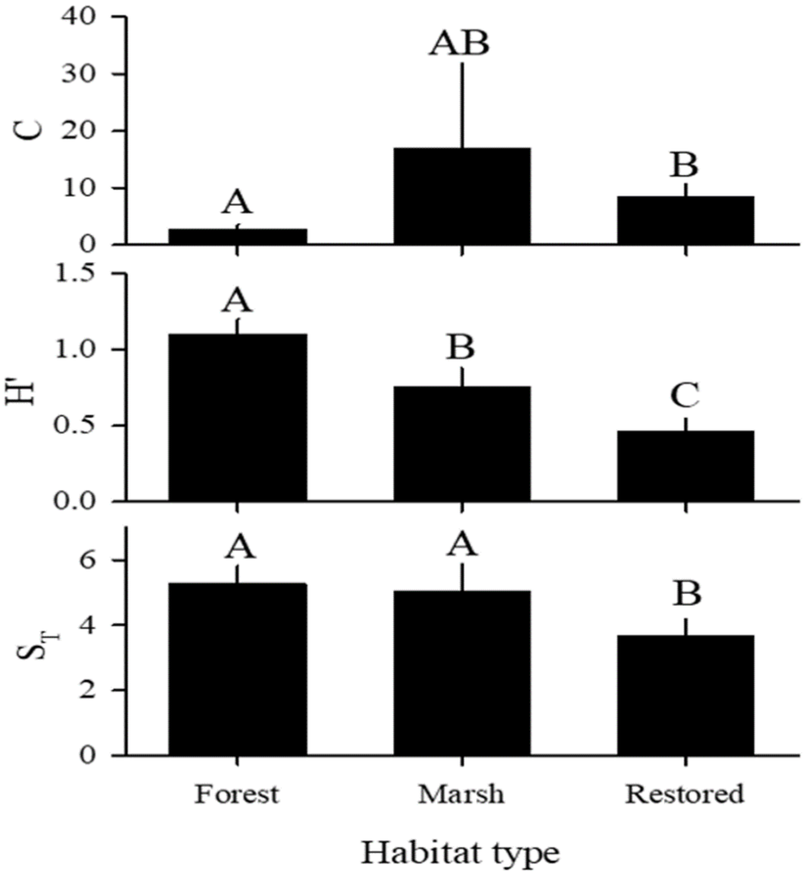

S2 Fig. Site comparisons. Mean concentration (ind/m^3), diversity (H'), and number of taxa (S_T) by habitat type. Letters denote results of ANOVA and SNK tests. Error bars are 95% confidence intervals.
