# Supplementary material for: Export of macroinvertebrate prey from tidal freshwater wetlands provides a significant energy subsidy for outmigrating juvenile salmon
Source: PLoS One. 2023 Mar 17;18(3):e0282655. doi: 10.1371/journal.pone.0282655 (PMC10022792; doi:10.1371/journal.pone.0282655)
Supplement: S3 Appendix — (PDF) [file pone.0282655.s003.pdf]

S3 Appendix. Regression analyses

We used regression to examine how the metrics *C, U, Q, F,* and T_I varied with bankfull area and stage of tide. Significant regressions would be evidence that these physical factors (channel size and hydraulic forcing) affected prey concentrations and rate metrics. We first examined planform allometry for each tidal creek, finding a close relationship of channel bankfull surface area (BA) and mouth width (S3 Fig A). We then used regression to evaluate if bankfull area was a predictor of the metrics *C, U, Q, F,* and T_I. We conducted two regression tests: station mean (mean of metrics per sample date) and grand mean (mean of all samples). Based on correlation coefficients, there was a poor fit to any relation except for a positive relation with discharge ($Q \times$ BA) (S3 Table; S3 Fig B). This conforms to the paradigm that larger systems had larger discharges, even as velocities were of similar range.

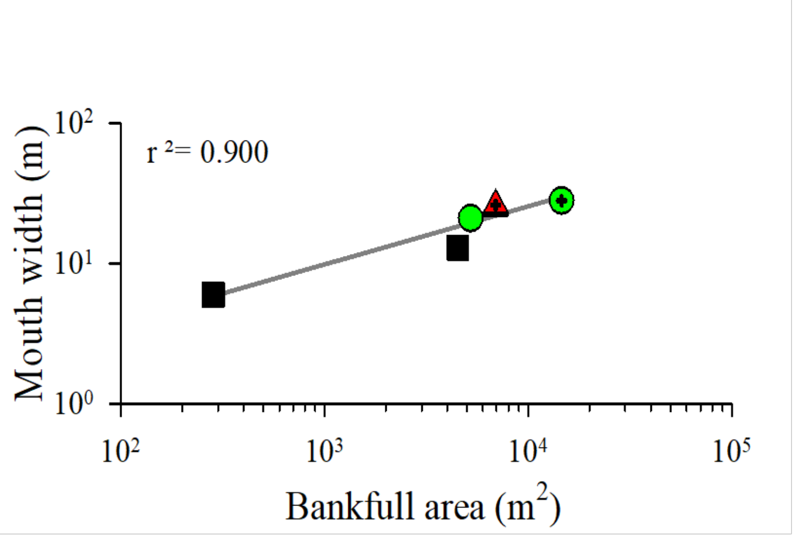

S3 Fig A. Hydraulic geometry for the six sampling sites in Cathlamet Bay. The emergent marsh sites. ME and MW had very similar geometries and points are superimposed on the plot.

S3 Table A. Regression parameters for concentration, flow, and flux metrics with bankfull area for six tidal creeks. Results of two tests are shown: station mean (mean of metrics per sample date) and grand mean (mean of all samples). *C*, concentration (prey/m^3); *U*, velocity (m/s). *Q*, discharge (m^3/s), *F*, flux (prey/m^2/s), and T_I, instantaneous transport (prey/s).

Test	Metric	Intercept	Slope	r²
Station mean	*C*	0.575	0.023	0.001
	U	-1.218	0.041	0.045
	Q	-1.122	0.331	0.564
	F	-0.679	0.061	0.005
	T_I	-0.465	0.298	0.123
Grand mean	*C*	0.632	0.010	<0.001
	U	-1.230	0.050	0.122
	Q	-1.192	0.360	0.857
	F	-0.533	0.031	0.002
	T_I	-0.427	0.311	0.214

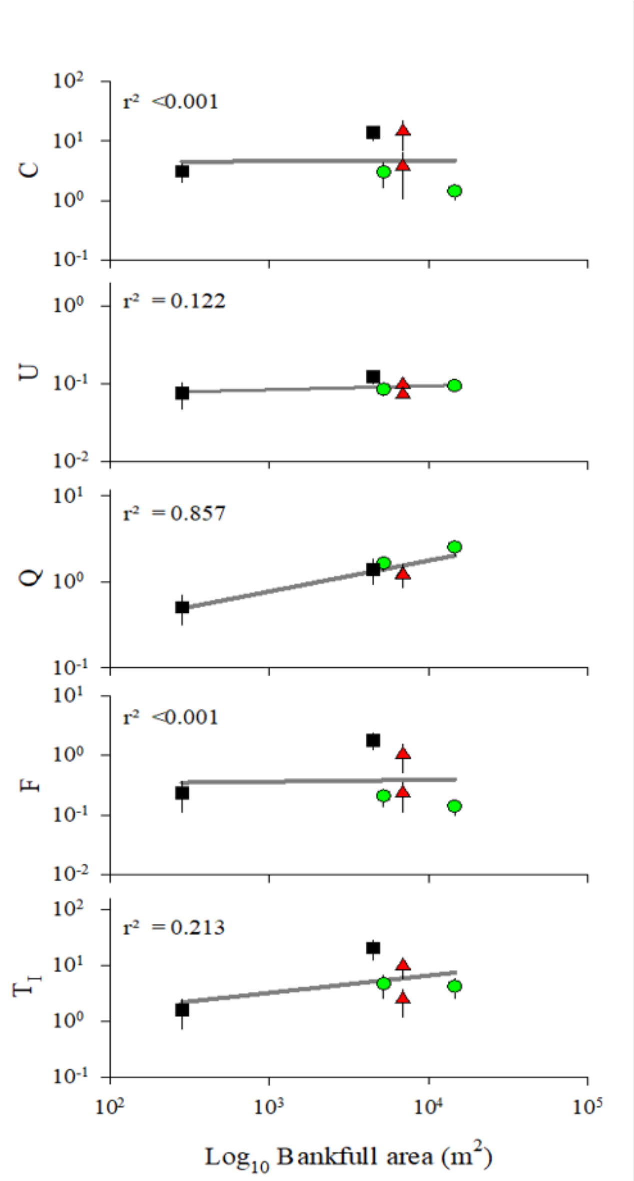

S3 Fig B. Regression plots. Regression of mean concentration, flow, and flux metrics with stage of tide. *C*, concentration (ind/m^3) for each habitat type. *C*, concentration (ind/m^3); *U*, velocity (m/s). *Q*, discharge (m^3/s), *F*, flux (ind/m^2/s), and T_I, instantaneous transport (prey/s). Data are mean measurements from all sample dates. Linear regression parameters are provided in S3 Table.

We then examined if stage of tide (hydraulic forcing) was a predictor of prey concentrations and rate metrics. Stage of tide was normalized to high tide as WSE/WSE_{MAX}, where WSE_{MAX} is the water surface elevation at high tide (1.00). We ran separate linear regressions (log-normal) for each habitat type. Based on correlation coefficients, there was a poor fit to most relations, with moderate r^2 for $Q \times SOT$ at forest and march habitats, where Q was negatively related to water elevation (S3 Table B, S3 Fig C). This

conforms to decreasing Q with decreasing cross-sectional areas of the tidal channels with the falling tide. Other metrics generally exhibit wide scatter without systematic patterns. For regressions with stage of tide, we standardize total prey concentration as C/C_{MAX} and the stage of tide as WSE/WSE_{MAX} (n=150 total).

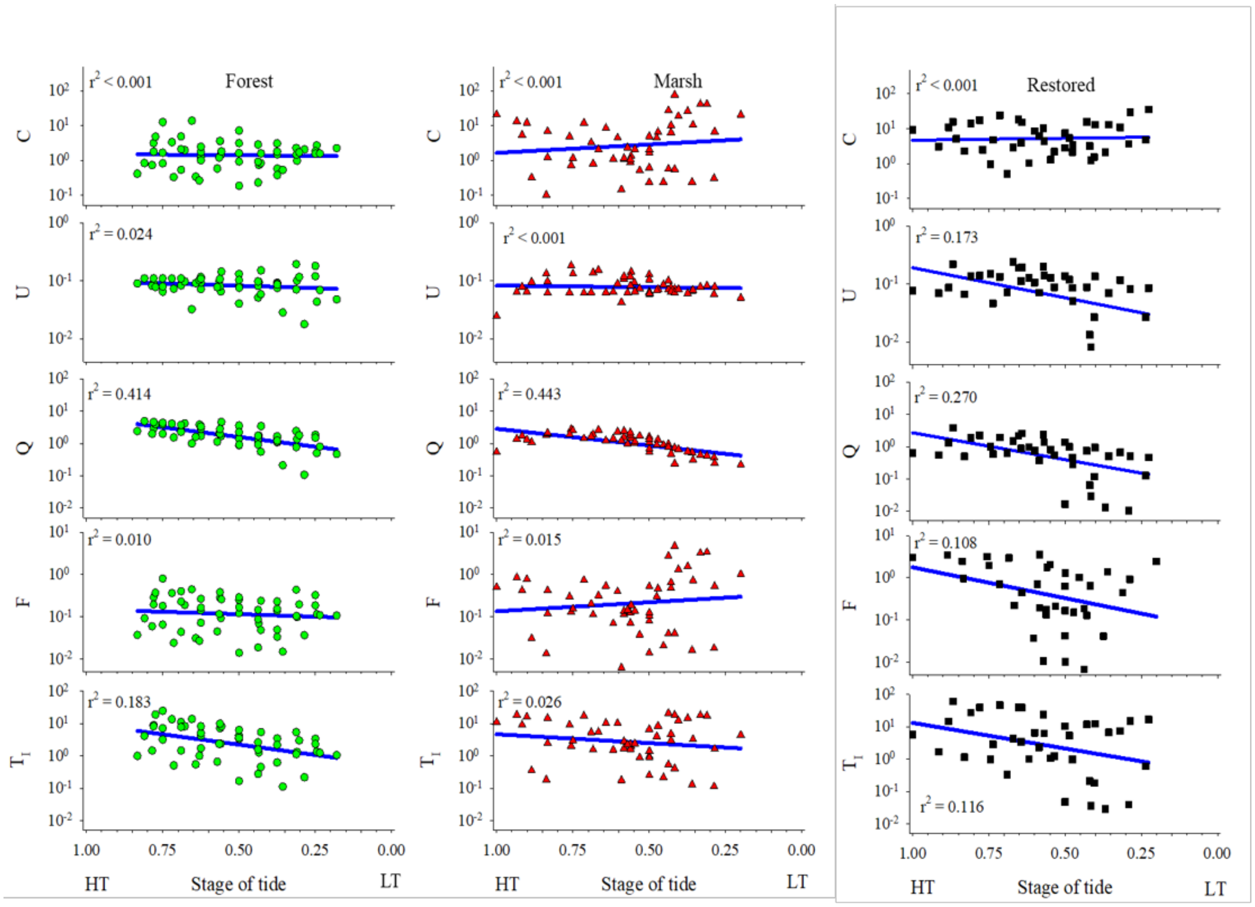

S3 Fig C. Regression of concentration, flow, and flux metrics with stage of tide. ***C*****, concentration (prey/m³) for each habitat type. C, concentration (prey/m³);** ***U*****, velocity (m/s).** ***Q*****, discharge (m³/s),** ***F*****, flux (prey/m²/s), and** ***T_I*****, instantaneous transport (prey/s). HT and LT, high and low tide, respectively. Data are individual measurements from all sample dates. Linear regression parameters are provided in S3 Table B.**

S3 Table B. Regression parameters for concentration, flow, and flux metrics with stage of tide. C_T, **concentration (ind/m^3);** U, **velocity (m/s).** Q, **discharge (m^3/s),** F_I, **instantaneous flux (ind/m^2/s), and** T_I, **instantaneous transport (ind/s).**

Metric	Habitat	Intercept	slope	r^2
C_T	Forest	0.112	0.073	0.001
	Marsh	0.701	-0.485	0.017
	Restor	0.785	-0.114	0.003
	All	0.424	-0.037	<0.001
U	Forest	0.161	-1.165	0.025
	Marsh	0.056	-1.138	0.005
	Restored	1.034	-1.753	0.174
	All	0.363	-1.313	0.054
Q	Forest	1.200	-0.399	0.415
	Marsh	1.032	-0.578	0.443
	Restored	1.667	-1.239	0.271
	All	1.165	-0.630	0.216
F_I	Forest	0.233	-1.054	0.010
	Marsh	-0.429	-0.439	0.015
	Restored	1.457	-1.211	0.108
	All	0.307	-0.881	0.008
T_I	Forest	1.273	-0.287	0.183
	Marsh	0.548	0.122	0.026
	Restored	1.588	-0.467	0.116
	All	1.103	-0.197	0.092

In this examination of whether tidal stage and channel size were predictive of prey concentration and flow metrics, we hypothesized fluxes might be highest during the early ebb phase of channel flow when velocities tend to be higher, as determined from our unpublished long-term ADCP measurements. But both the present study and these longer deployment data indicate that most velocities after this initial period were similar and relatively low (< 0.1 m/s) across both tidal stage and sites (S3 Figs B and C). Prey concentrations were also unrelated to stage of tide, and exhibited relatively high variation across sample times within a date (S3 Table B; S3 Fig C). Standardized concentrations were likewise poorly correlated with stage of tide, indicating maximum concentrations exhibited no consistent pattern with stage (S3 Fig D). Since fluxes are the product of velocity and concentration, this variability resulted in weak trends over tidal time periods. The same argument holds for instantaneous transport. Together, the data indicated little predictability in the timing of prey flux or transport by tide stage.

Bankfull area was also a poor predictor of mean prey concentration, velocity, or flux over the tidal ranges sampled (S3 Fig D). Mean velocities also did not scale with system size. We measured a maximum channel flow velocity of about 0.3 m/s for the hydraulic geometries of these systems, which is important for functions such as sedimentary processes, fish swimming behaviors, and the absolute magnitude of material fluxes. Discharge over the range of 10^4 to 10^6 m^3/tide was positively correlated with bankfull area. Larger wetland systems generally have larger watersheds and wider channel mouth cross-sectional areas [16], which results in higher discharges. Integrating an identical flux rate from different sized tidal creeks will always yield higher discharge in the larger geometry. However, the high variability in concentrations reduced the strength of the relationship with instantaneous measures of transport.

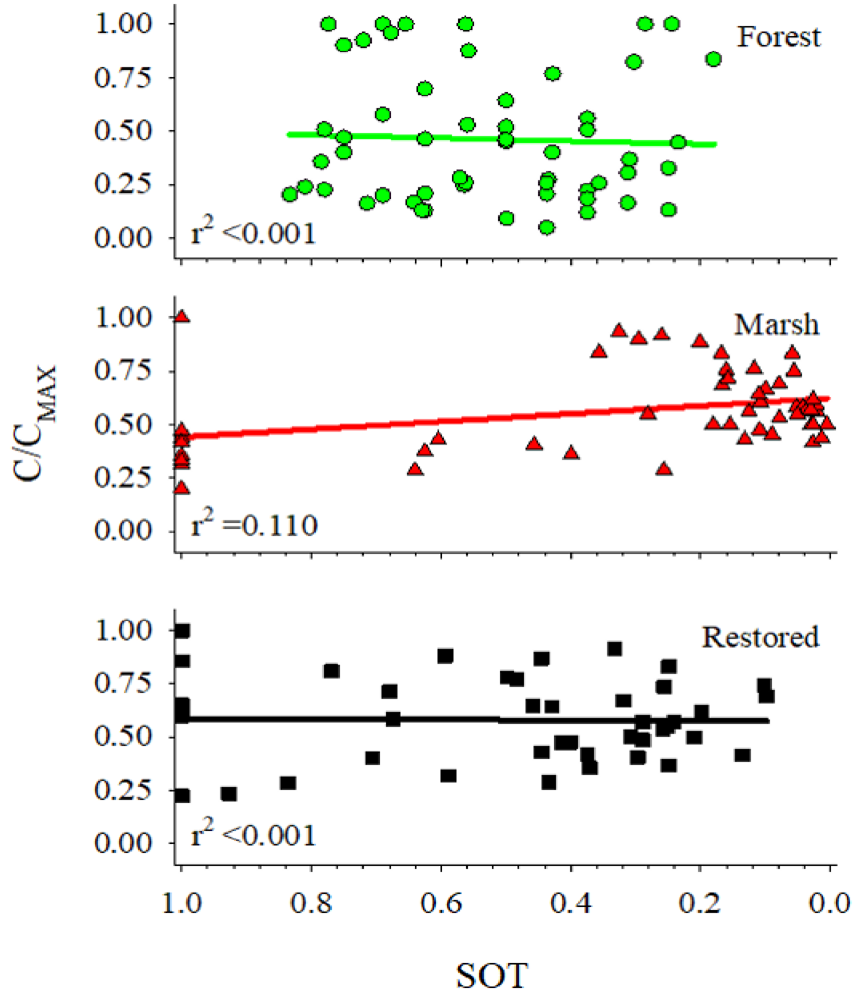

S3 Fig D. Regression of normalized concentration (C/C_{MAX}) with stage of tide (SOT) for each habitat type. HT and LT, high and low tide, respectively. Data are individual measurements from all sample dates. Linear regression parameters are provided in S3 Table C.

S3 Table C. Regression parameters for normalized concentration (C_T/C_{MAX}) with stage of tide.

Metric	Habitat	Intercept	Slope	r^2
C_T/C_{MAX}	Forest	0.423	0.074	<0.001
	Marsh	0.624	-0.183	0.109
	Restor	0.575	0.011	<0.001
